# Supplementary material for: Enalapril mitigates senescence and aging-related phenotypes in human cells and mice via pSmad1/5/9-driven antioxidative genes
Source: eLife. 2025 Aug 28;14:RP104774. doi: 10.7554/eLife.104774 (PMC12393883; doi:10.7554/eLife.104774)
Supplement: Figure 1—source data 1. [file elife-104774-fig1-data1.zip › Figure1-source data1/Figure1-source data1.pdf]

Figure 1, Source Data 1

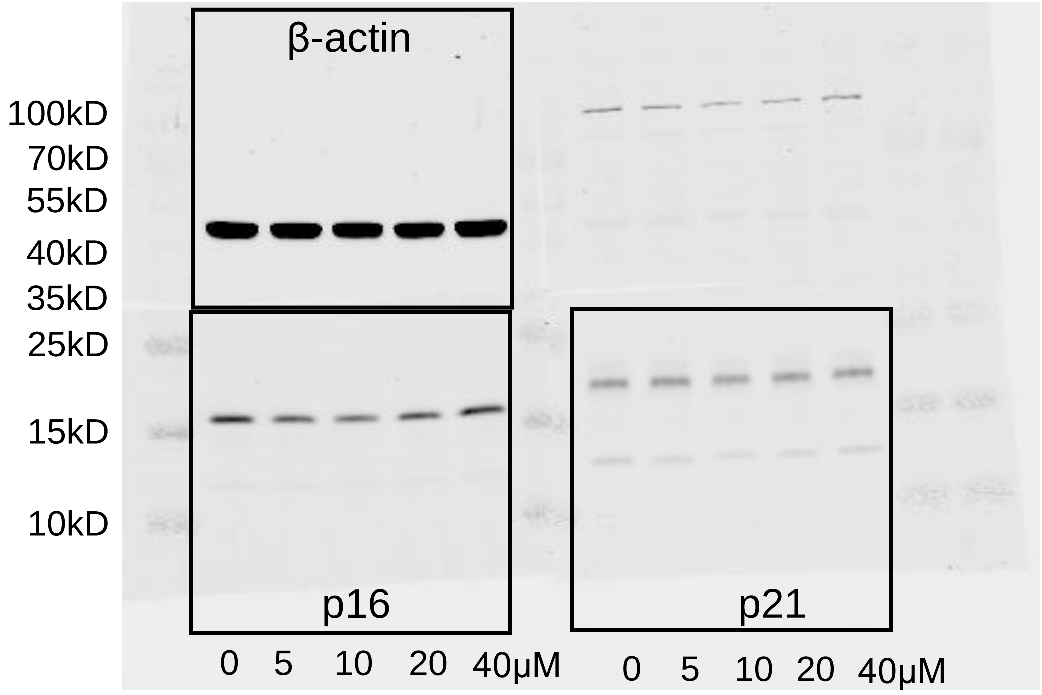

Figure 1, Source Data 1. Original membranes corresponding to Figure 1C. Lanes from left to right correspond to IMR90 cells treated with enalapril at 0, 5, 10, 20, and 40 μM, respectively.
