# Supplementary figures and images for: Enalapril mitigates senescence and aging-related phenotypes in human cells and mice via pSmad1/5/9-driven antioxidative genes
Source: eLife. 2025 Aug 28;14:RP104774. doi: 10.7554/eLife.104774 (PMC12393883; doi:10.7554/eLife.104774)

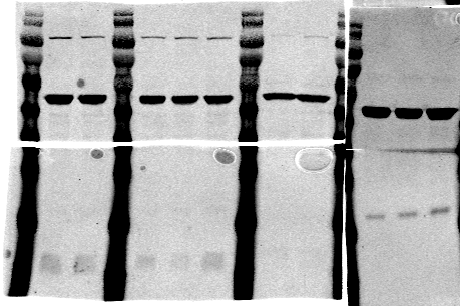

Supplement: Figure 1—figure supplement 1—source data 2. [file elife-104774-fig1-figsupp1-data2.zip › Figure1-figure supplement1-source data2/p16.tif]

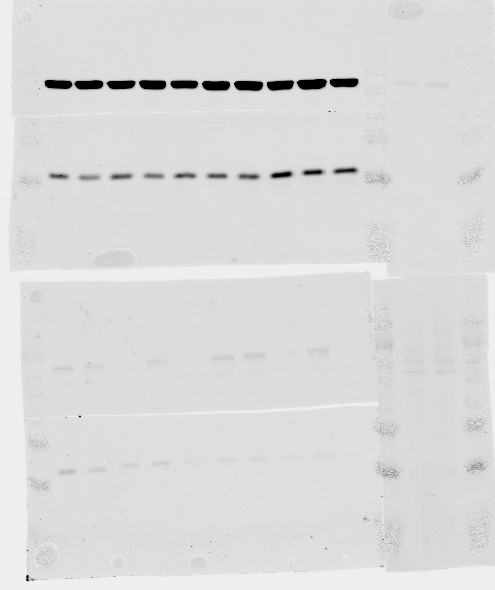

Supplement: Figure 2—source data 2. [file elife-104774-fig2-data2.zip › Figure2-source data2/p16, ╬▓-actin.tif]

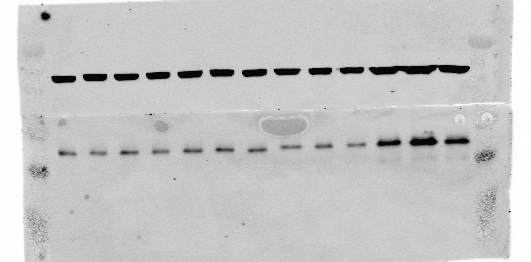

Supplement: Figure 2—source data 2. [file elife-104774-fig2-data2.zip › Figure2-source data2/p21.tif]

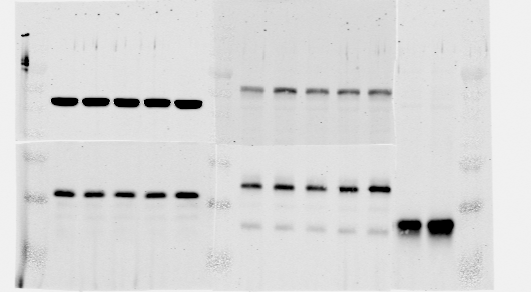

Supplement: Figure 2—source data 2. [file elife-104774-fig2-data2.zip › Figure2-source data2/pSmad159-A.tif]

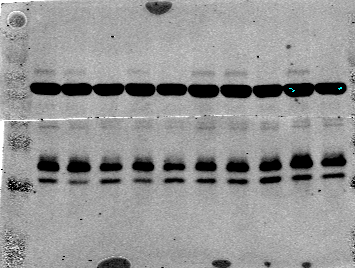

Supplement: Figure 2—source data 2. [file elife-104774-fig2-data2.zip › Figure2-source data2/pSmad159-E.tif]

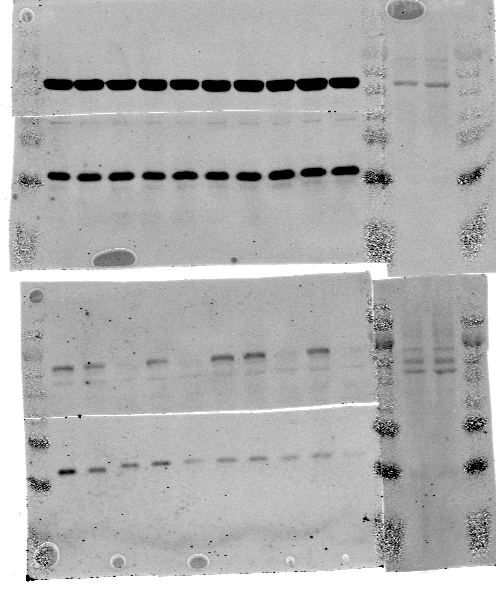

Supplement: Figure 2—source data 2. [file elife-104774-fig2-data2.zip › Figure2-source data2/pSmad2, pSmad3.tif]

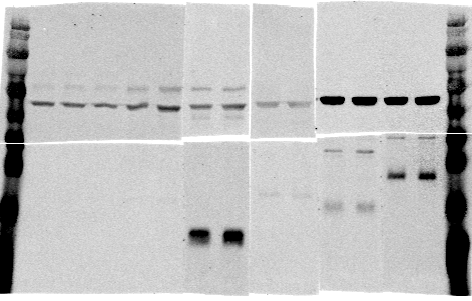

Supplement: Figure 2—source data 2. [file elife-104774-fig2-data2.zip › Figure2-source data2/pSmad5, ╬▓-actin.tif]

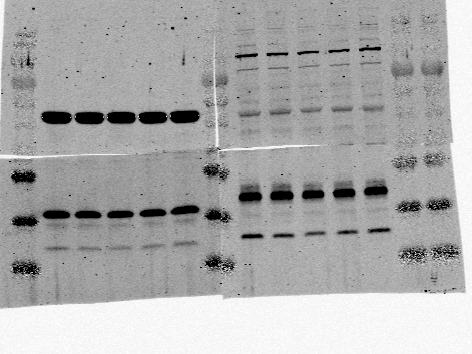

Supplement: Figure 2—source data 2. [file elife-104774-fig2-data2.zip › Figure2-source data2/Smad159-A.tif]

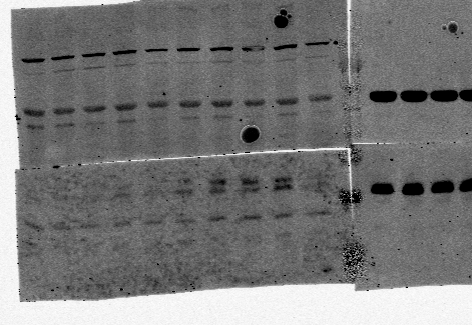

Supplement: Figure 2—source data 2. [file elife-104774-fig2-data2.zip › Figure2-source data2/Smad159-E.tif]

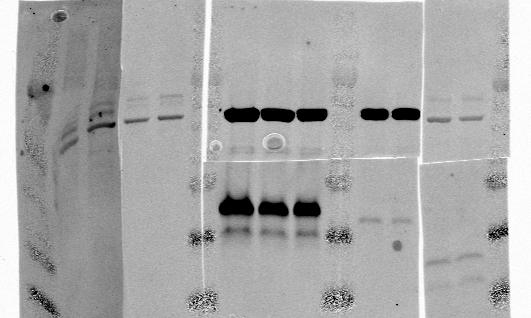

Supplement: Figure 2—source data 2. [file elife-104774-fig2-data2.zip › Figure2-source data2/Smad23.tif]

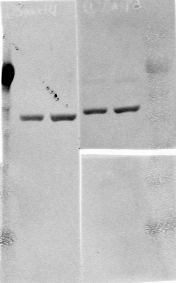

Supplement: Figure 2—source data 2. [file elife-104774-fig2-data2.zip › Figure2-source data2/Smad4.tif]

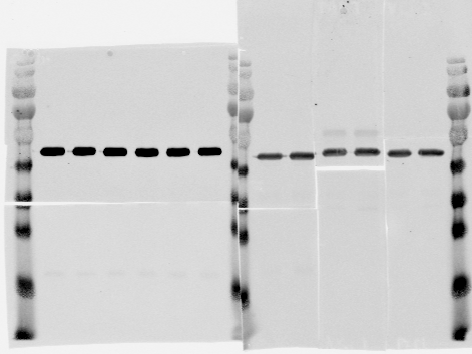

Supplement: Figure 2—figure supplement 1—source data 2. [file elife-104774-fig2-figsupp1-data2.zip › Figure2-figure supplement1-source data2/pAKT, pERK.tif]

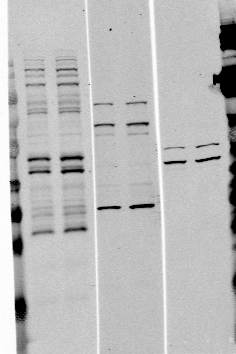

Supplement: Figure 2—figure supplement 1—source data 2. [file elife-104774-fig2-figsupp1-data2.zip › Figure2-figure supplement1-source data2/pmTOR, AKT, ERK.tif]

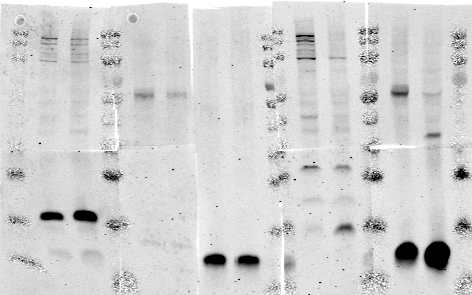

Supplement: Figure 2—figure supplement 1—source data 2. [file elife-104774-fig2-figsupp1-data2.zip › Figure2-figure supplement1-source data2/pSmad159.tif]

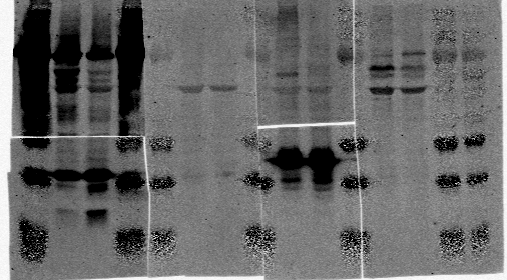

Supplement: Figure 2—figure supplement 1—source data 2. [file elife-104774-fig2-figsupp1-data2.zip › Figure2-figure supplement1-source data2/pSmad2, pSmad3, Smad23.tif]

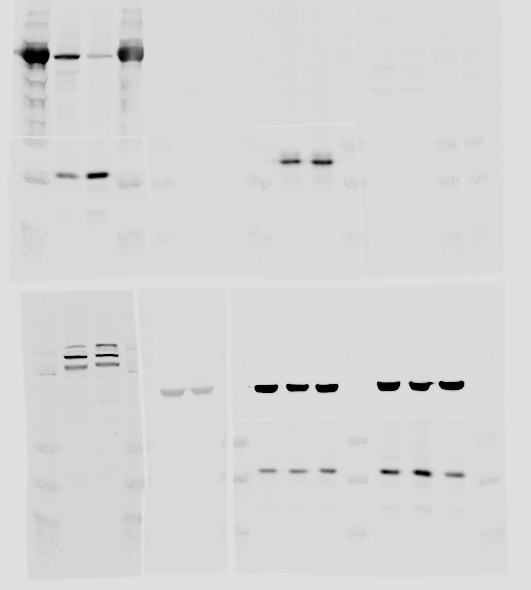

Supplement: Figure 2—figure supplement 1—source data 2. [file elife-104774-fig2-figsupp1-data2.zip › Figure2-figure supplement1-source data2/Smad4, p16, p21, BMP2, BMP4.tif]

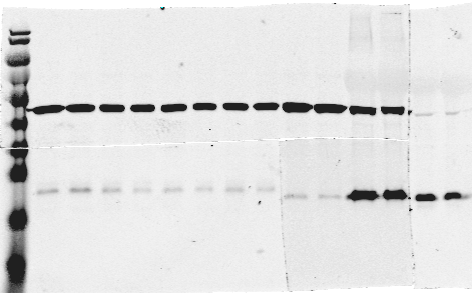

Supplement: Figure 2—figure supplement 1—source data 2. [file elife-104774-fig2-figsupp1-data2.zip › Figure2-figure supplement1-source data2/╬▓-actin-A.tif]

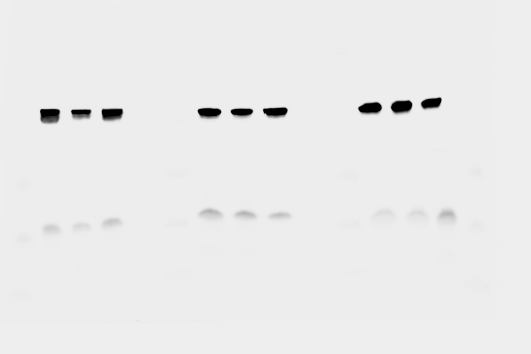

Supplement: Figure 2—figure supplement 1—source data 2. [file elife-104774-fig2-figsupp1-data2.zip › Figure2-figure supplement1-source data2/╬▓-actin-B.tif]

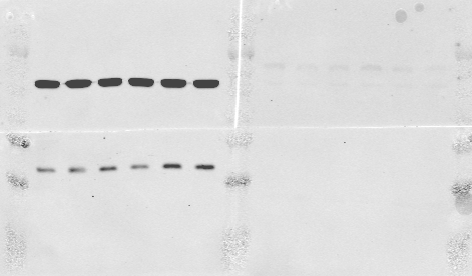

Supplement: Figure 2—figure supplement 2—source data 2. [file elife-104774-fig2-figsupp2-data2.zip › Figure2-figure supplement2-source data2/ID1.tif]

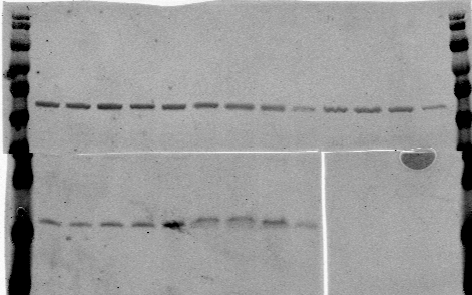

Supplement: Figure 2—figure supplement 2—source data 2. [file elife-104774-fig2-figsupp2-data2.zip › Figure2-figure supplement2-source data2/p16.tif]

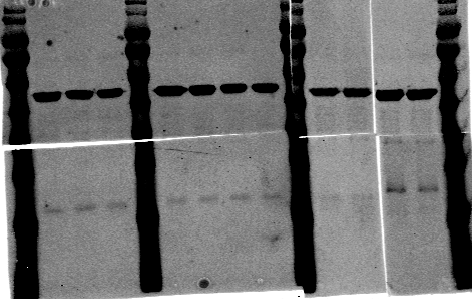

Supplement: Figure 2—figure supplement 2—source data 2. [file elife-104774-fig2-figsupp2-data2.zip › Figure2-figure supplement2-source data2/p21.tif]

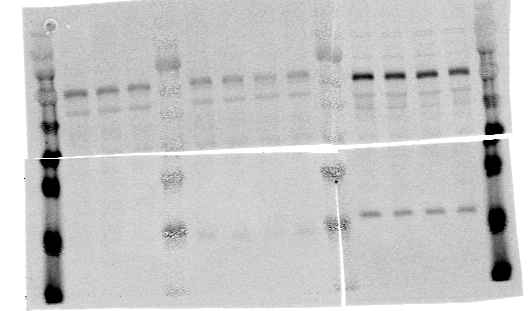

Supplement: Figure 2—figure supplement 2—source data 2. [file elife-104774-fig2-figsupp2-data2.zip › Figure2-figure supplement2-source data2/pSmad5, Smad5.tif]

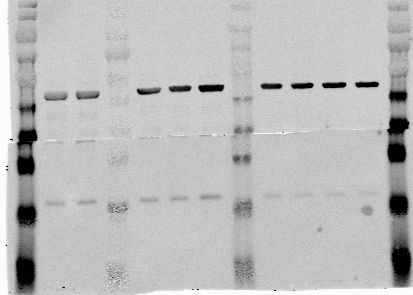

Supplement: Figure 2—figure supplement 2—source data 2. [file elife-104774-fig2-figsupp2-data2.zip › Figure2-figure supplement2-source data2/╬▓-actin.tif]

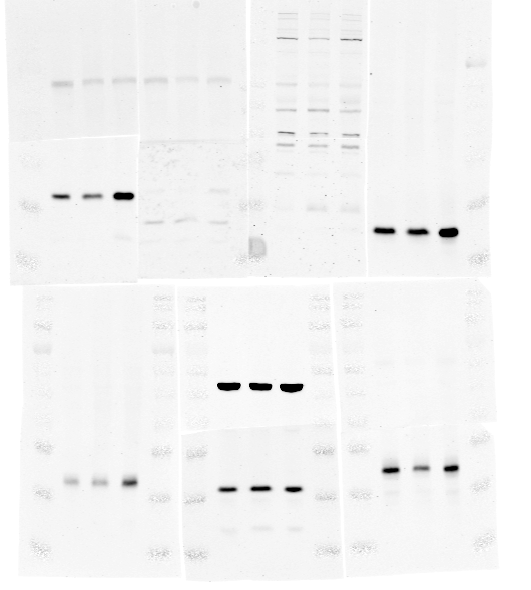

Supplement: Figure 2—figure supplement 3—source data 2. [file elife-104774-fig2-figsupp3-data2.zip › Figure2-figure supplement3-source data2/ID1.tif]

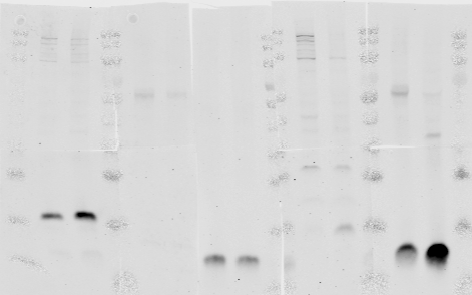

Supplement: Figure 2—figure supplement 3—source data 2. [file elife-104774-fig2-figsupp3-data2.zip › Figure2-figure supplement3-source data2/p16, TXN.tif]

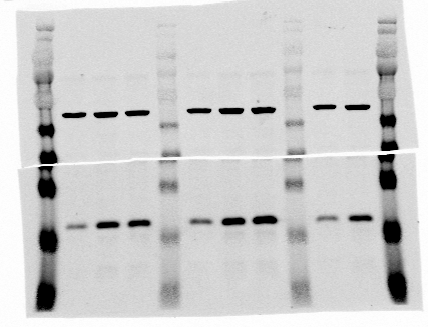

Supplement: Figure 2—figure supplement 3—source data 2. [file elife-104774-fig2-figsupp3-data2.zip › Figure2-figure supplement3-source data2/p16, ╬▓-actin.tif]

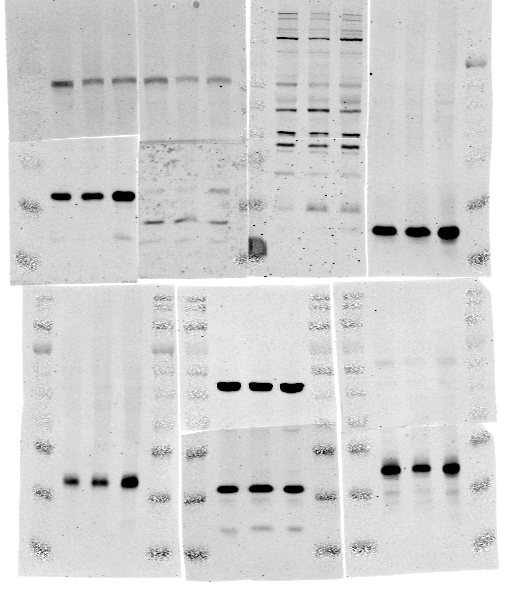

Supplement: Figure 2—figure supplement 3—source data 2. [file elife-104774-fig2-figsupp3-data2.zip › Figure2-figure supplement3-source data2/pSmad159, pSmad5, Smad159, PRDX5, ╬▓-actin.tif]

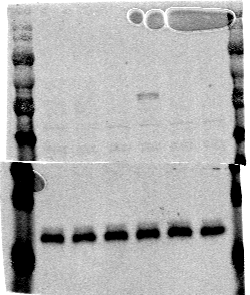

Supplement: Figure 2—figure supplement 3—source data 2. [file elife-104774-fig2-figsupp3-data2.zip › Figure2-figure supplement3-source data2/pSmad159.tif]

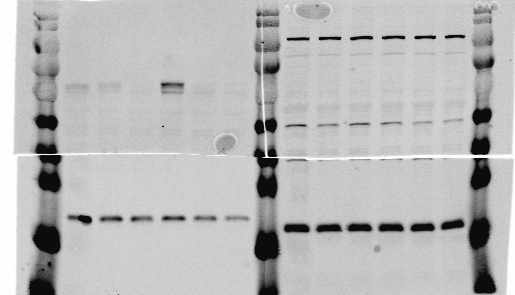

Supplement: Figure 2—figure supplement 3—source data 2. [file elife-104774-fig2-figsupp3-data2.zip › Figure2-figure supplement3-source data2/pSmad5, Smad159.tif]

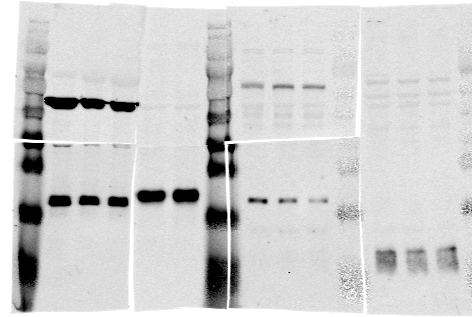

Supplement: Figure 2—figure supplement 3—source data 2. [file elife-104774-fig2-figsupp3-data2.zip › Figure2-figure supplement3-source data2/Smad5.tif]

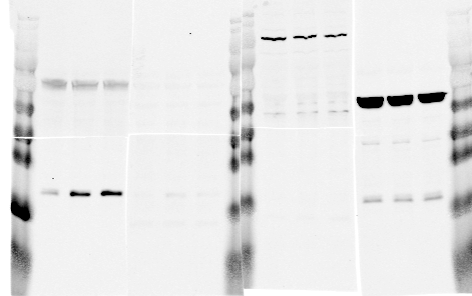

Supplement: Figure 3—source data 2. [file elife-104774-fig3-data2.zip › Figure3-source data2/ID1, ╬▓-actin-C.tif]

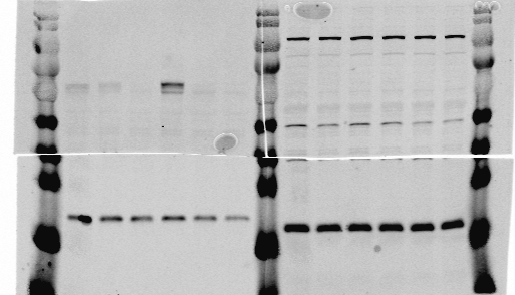

Supplement: Figure 3—source data 2. [file elife-104774-fig3-data2.zip › Figure3-source data2/ID1-E.tif]

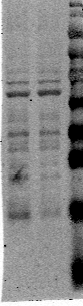

Supplement: Figure 3—source data 2. [file elife-104774-fig3-data2.zip › Figure3-source data2/ID1-F.tif]

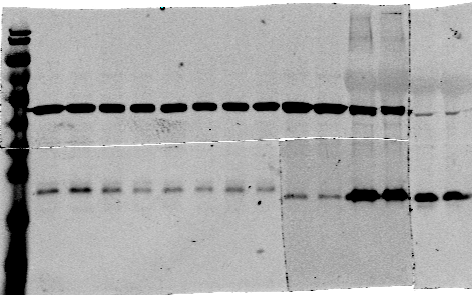

Supplement: Figure 3—source data 2. [file elife-104774-fig3-data2.zip › Figure3-source data2/ID1-I.tif]

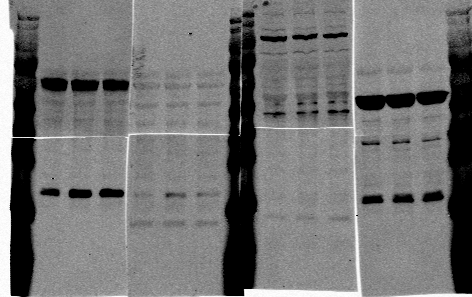

Supplement: Figure 3—source data 2. [file elife-104774-fig3-data2.zip › Figure3-source data2/ID2-C.tif]

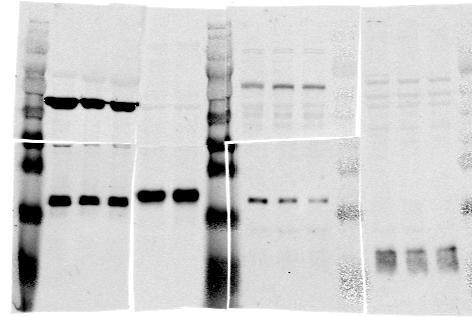

Supplement: Figure 3—source data 2. [file elife-104774-fig3-data2.zip › Figure3-source data2/ID2-E.tif]

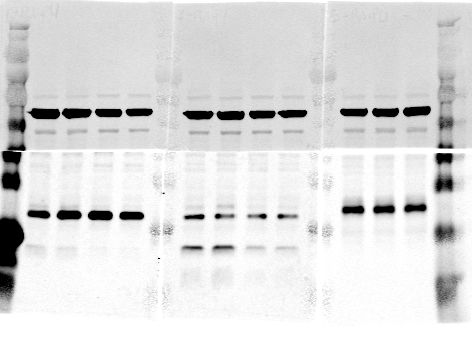

Supplement: Figure 3—source data 2. [file elife-104774-fig3-data2.zip › Figure3-source data2/ID2-F.tif]

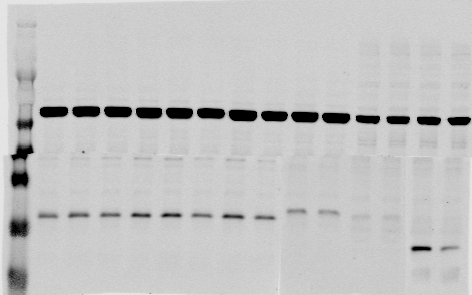

Supplement: Figure 3—source data 2. [file elife-104774-fig3-data2.zip › Figure3-source data2/p16, ╬▓-actin-F.tif]

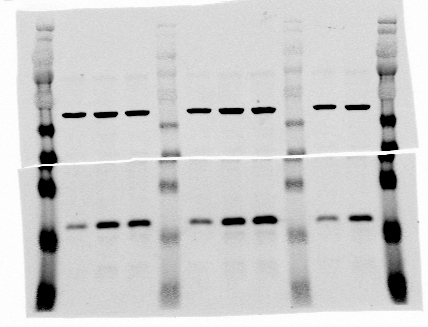

Supplement: Figure 3—source data 2. [file elife-104774-fig3-data2.zip › Figure3-source data2/p16-I.tif]

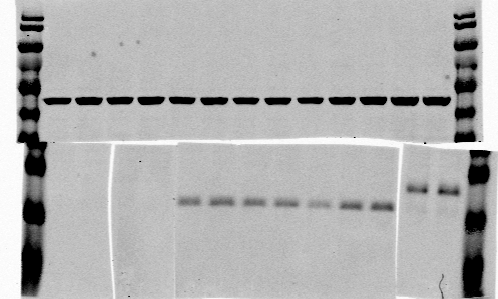

Supplement: Figure 3—source data 2. [file elife-104774-fig3-data2.zip › Figure3-source data2/p21, ╬▓-actin-I.tif]

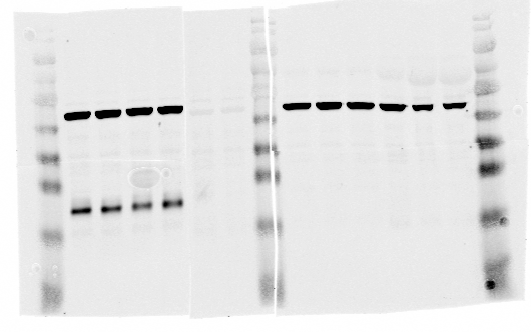

Supplement: Figure 3—source data 2. [file elife-104774-fig3-data2.zip › Figure3-source data2/p21-ID1KD-F.tif]

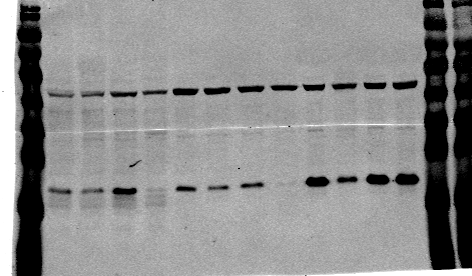

Supplement: Figure 3—source data 2. [file elife-104774-fig3-data2.zip › Figure3-source data2/p21-ID2KD-F.tif]

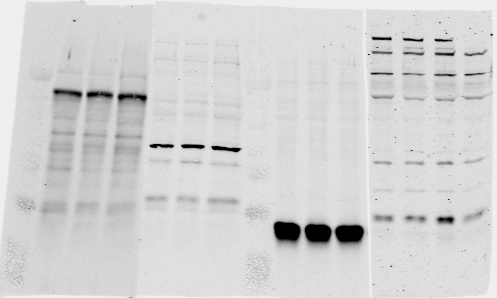

Supplement: Figure 3—source data 2. [file elife-104774-fig3-data2.zip › Figure3-source data2/pSmad159, Smad159-J.tif]

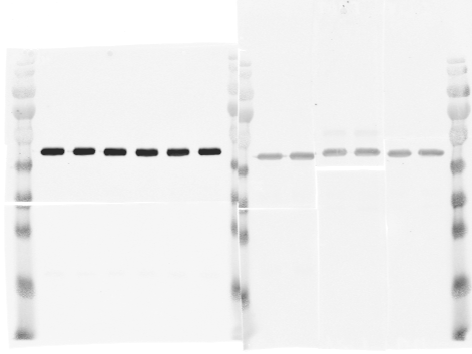

Supplement: Figure 3—source data 2. [file elife-104774-fig3-data2.zip › Figure3-source data2/╬▓-actin-E.tif]

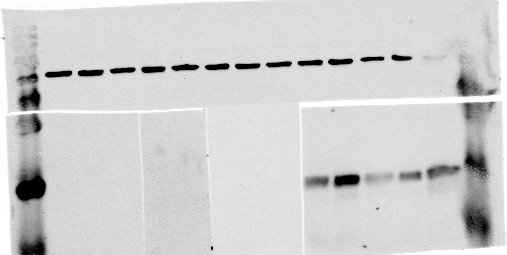

Supplement: Figure 3—source data 2. [file elife-104774-fig3-data2.zip › Figure3-source data2/╬▓-actin-J.tif]

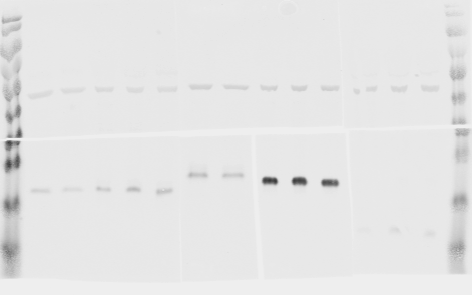

Supplement: Figure 3—figure supplement 1—source data 2. [file elife-104774-fig3-figsupp1-data2.zip › Figure3-figure supplement1-source data2/ID1, ╬▓-actin.tif]

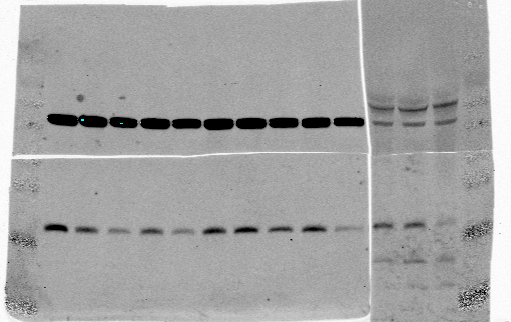

Supplement: Figure 3—figure supplement 1—source data 2. [file elife-104774-fig3-figsupp1-data2.zip › Figure3-figure supplement1-source data2/ID2.tif]

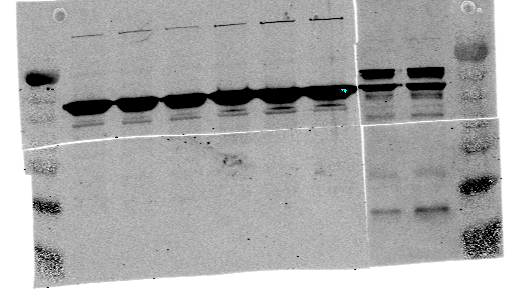

Supplement: Figure 4—source data 2. [file elife-104774-fig4-data2.zip › Figure4-source data2/GPX4.tif]

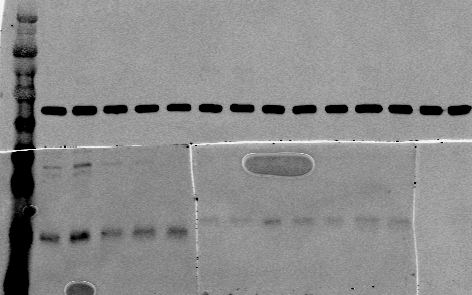

Supplement: Figure 4—source data 2. [file elife-104774-fig4-data2.zip › Figure4-source data2/PRDX5.tif]

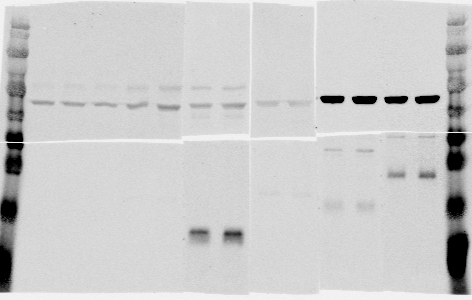

Supplement: Figure 4—source data 2. [file elife-104774-fig4-data2.zip › Figure4-source data2/TXN, NDUFB10, ╬▓-actin.tif]

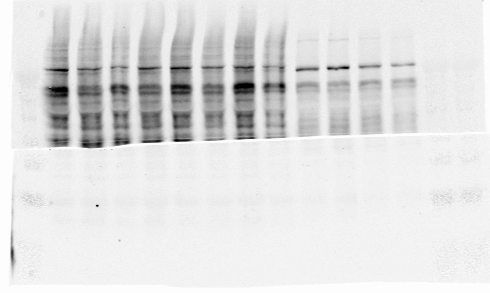

Supplement: Figure 5—source data 2. [file elife-104774-fig5-data2.zip › Figure5-source data2/pSmad159-spleen.tif]

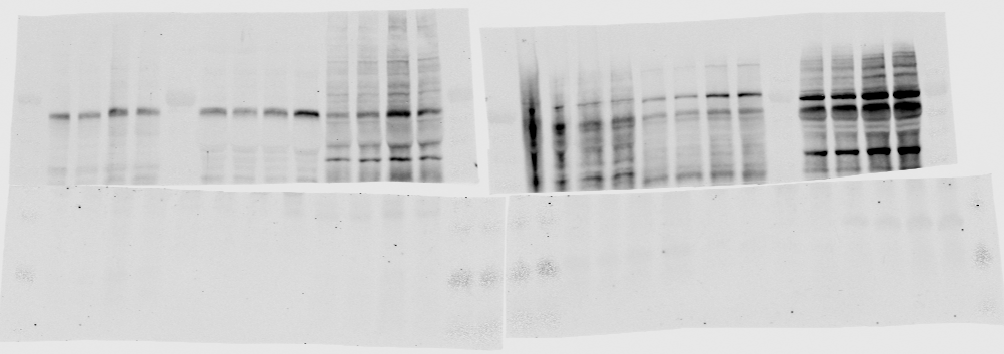

Supplement: Figure 5—source data 2. [file elife-104774-fig5-data2.zip › Figure5-source data2/pSmad159.tif]

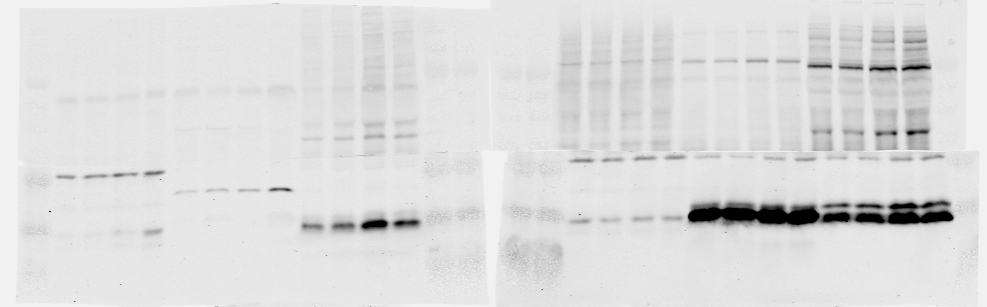

Supplement: Figure 5—source data 2. [file elife-104774-fig5-data2.zip › Figure5-source data2/pSmad5, Prdx5.tif]

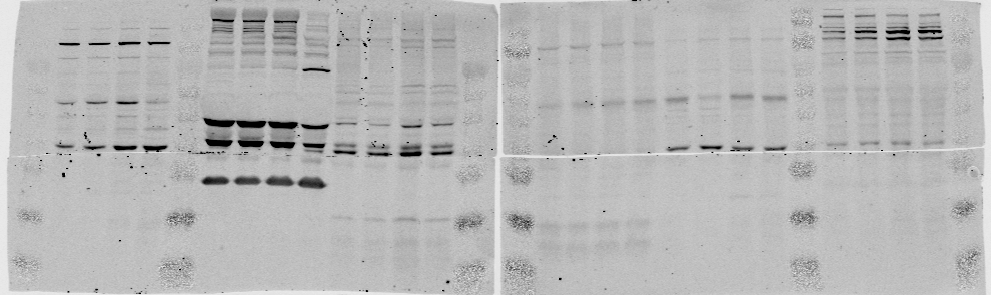

Supplement: Figure 5—source data 2. [file elife-104774-fig5-data2.zip › Figure5-source data2/Smad159.tif]

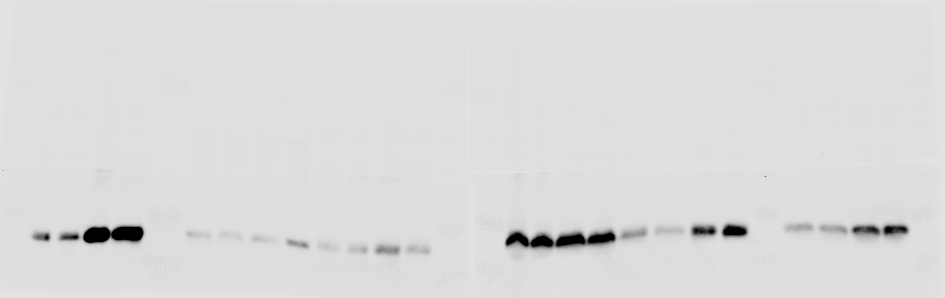

Supplement: Figure 5—source data 2. [file elife-104774-fig5-data2.zip › Figure5-source data2/Txn1.tif]

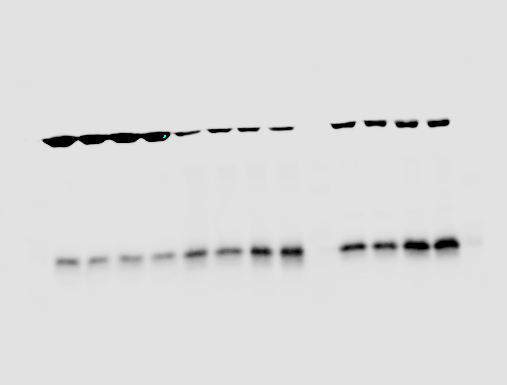

Supplement: Figure 5—source data 2. [file elife-104774-fig5-data2.zip › Figure5-source data2/Txn2, ╬▓-actin.tif]

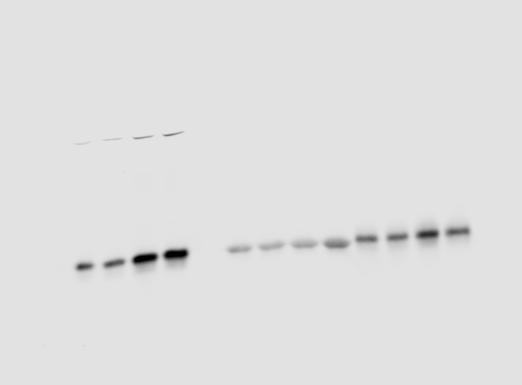

Supplement: Figure 5—source data 2. [file elife-104774-fig5-data2.zip › Figure5-source data2/Txn2.tif]

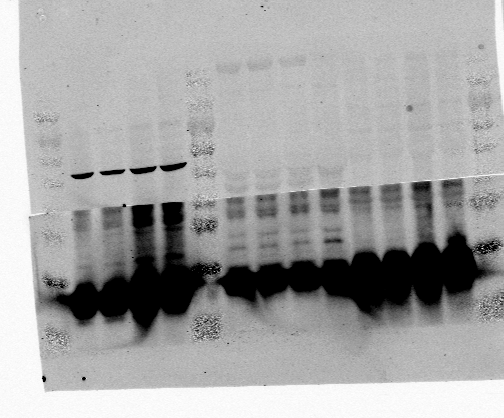

Supplement: Figure 5—source data 2. [file elife-104774-fig5-data2.zip › Figure5-source data2/╬▓-actin-liver.tif]

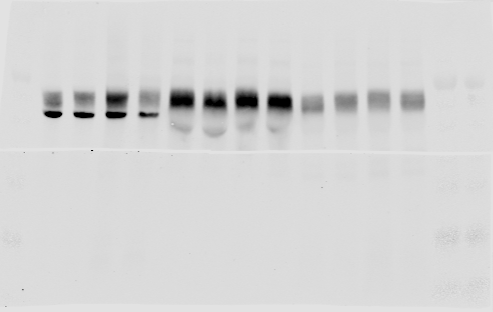

Supplement: Figure 5—source data 2. [file elife-104774-fig5-data2.zip › Figure5-source data2/╬▓-actin-muscle, heart.tif]

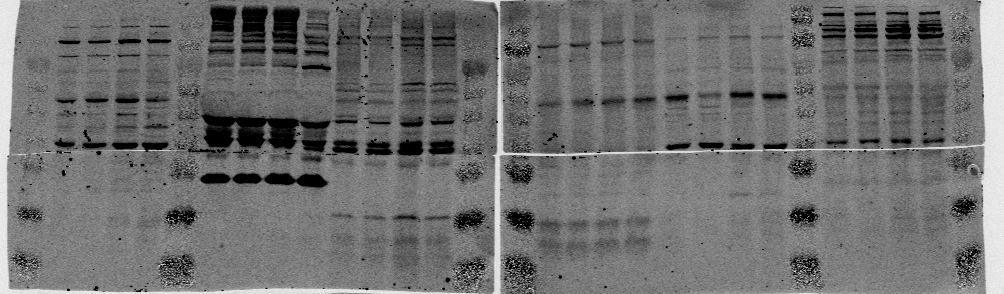

Supplement: Figure 5—figure supplement 1—source data 2. [file elife-104774-fig5-figsupp1-data2.zip › Figure5-figure supplement1-source data2/Id1.tif]

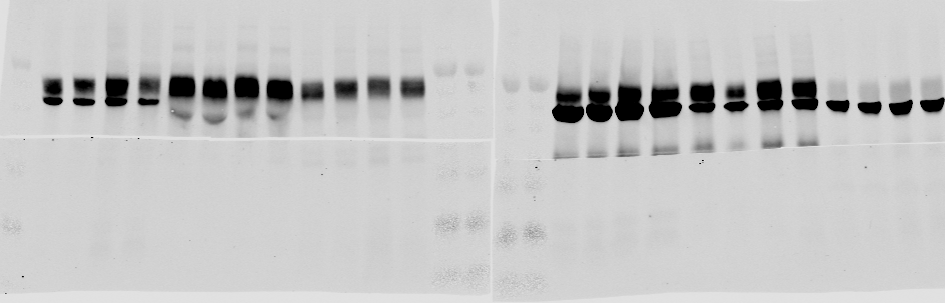

Supplement: Figure 5—figure supplement 1—source data 2. [file elife-104774-fig5-figsupp1-data2.zip › Figure5-figure supplement1-source data2/╬▓-actin.tif]

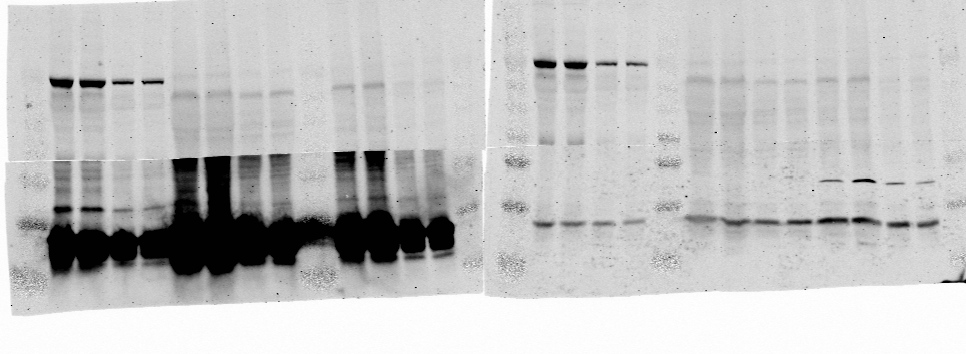

Supplement: Figure 5—figure supplement 2—source data 2. [file elife-104774-fig5-figsupp2-data2.zip › Figure5-figure supplement2-source data2/pSmad5, pSmad159.tif]

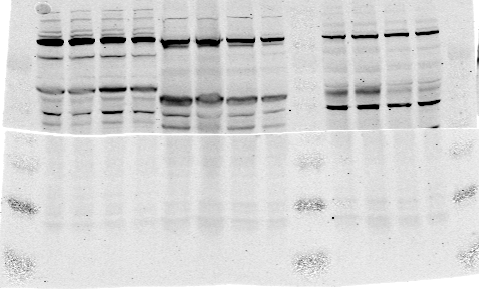

Supplement: Figure 5—figure supplement 2—source data 2. [file elife-104774-fig5-figsupp2-data2.zip › Figure5-figure supplement2-source data2/Smad159.tif]

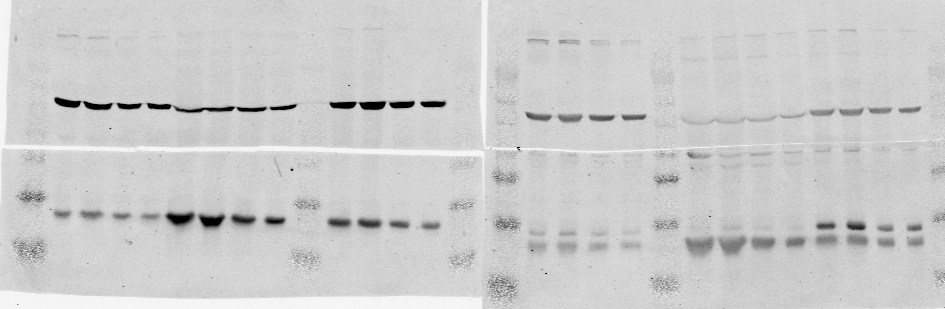

Supplement: Figure 5—figure supplement 2—source data 2. [file elife-104774-fig5-figsupp2-data2.zip › Figure5-figure supplement2-source data2/Txn1, Prdx5, ╬▓-actin.tif]

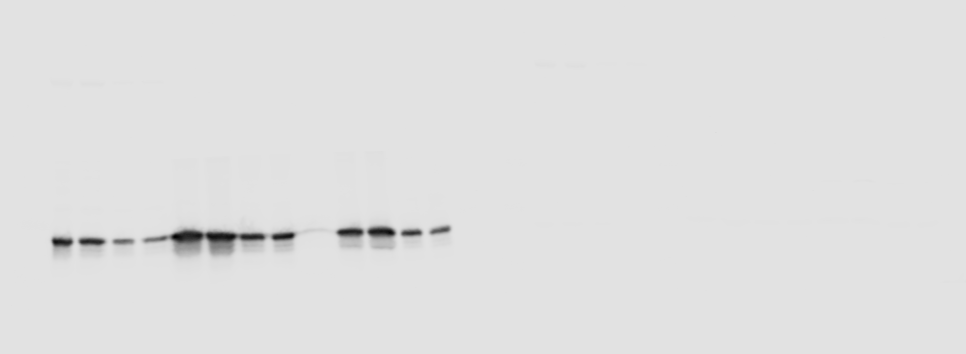

Supplement: Figure 5—figure supplement 2—source data 2. [file elife-104774-fig5-figsupp2-data2.zip › Figure5-figure supplement2-source data2/Txn2.tif]
