# Supplementary material for: Enalapril mitigates senescence and aging-related phenotypes in human cells and mice via pSmad1/5/9-driven antioxidative genes
Source: eLife. 2025 Aug 28;14:RP104774. doi: 10.7554/eLife.104774 (PMC12393883; doi:10.7554/eLife.104774)
Supplement: Figure 2—source data 1. [file elife-104774-fig2-data1.zip › Figure2-source data1/Figure2-source data1.pdf]

Figure 2, Source Data 1

Figure 2A

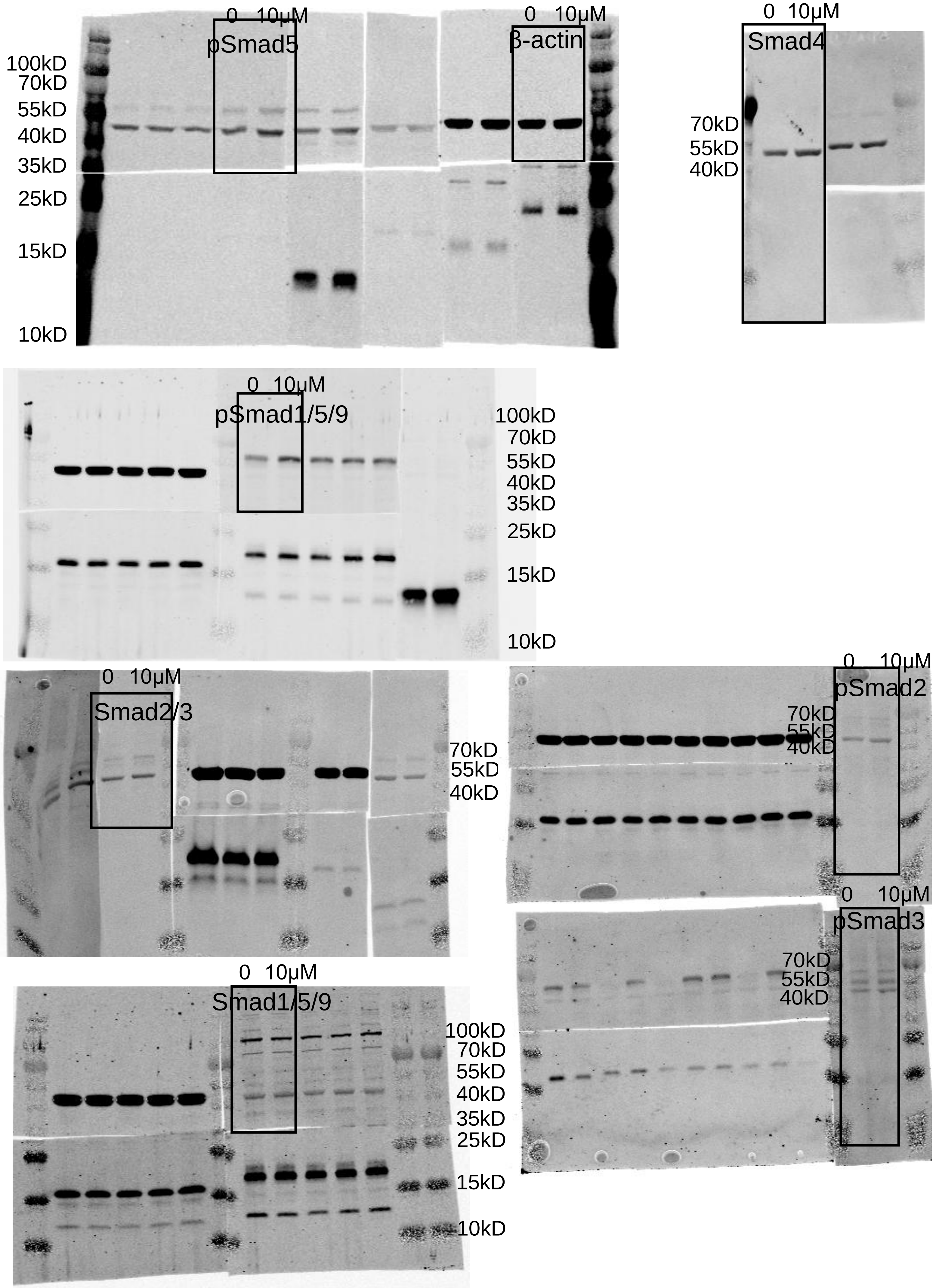

Figure 2, Source Data 1   Figure 2E

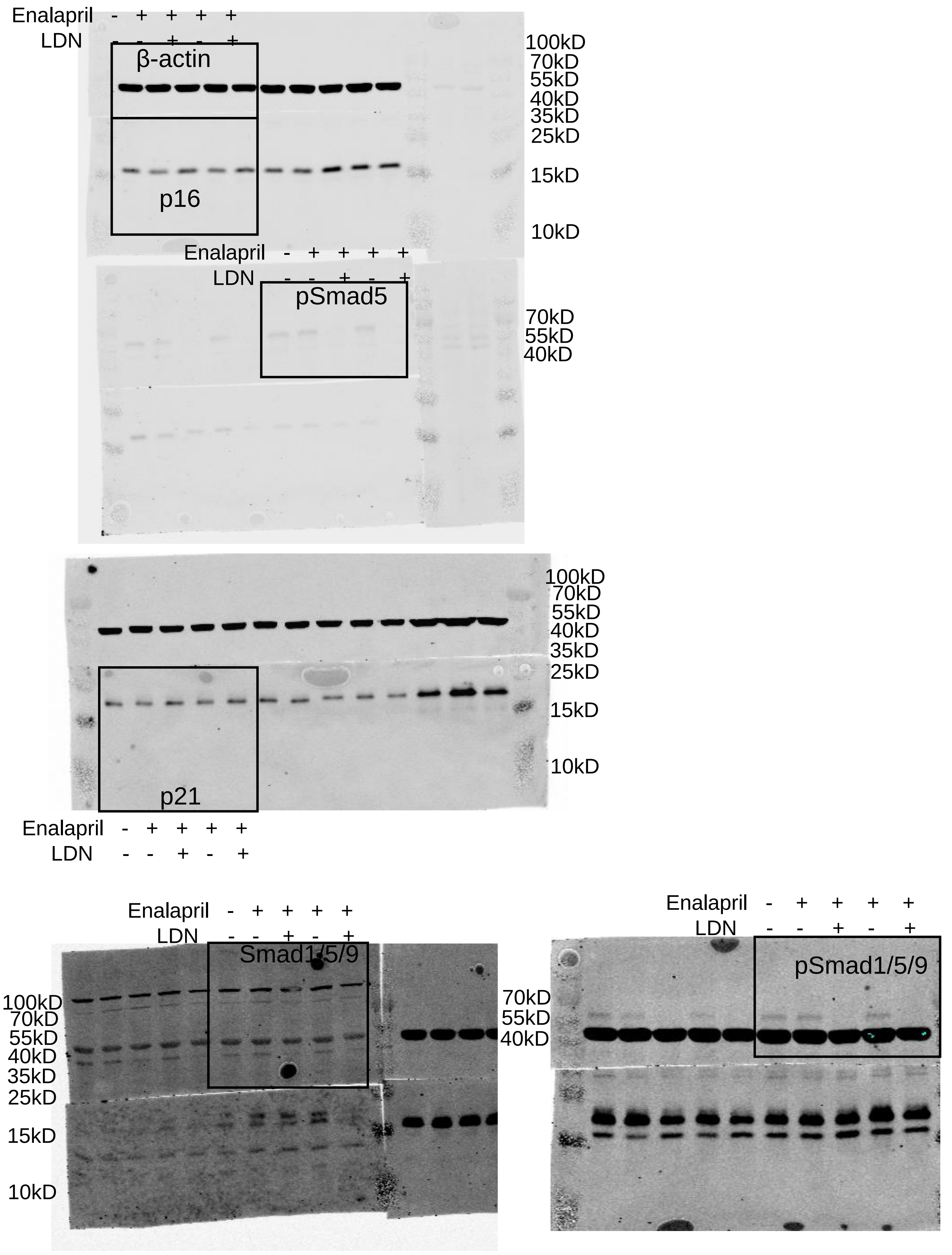

Figure 2, Source Data 1. Original membranes corresponding to Figure 2A, E. Lanes of Figure 2E from left to right correspond to IMR90 cells treated with enalapril at 0 and 10μM, respectively. Lanes of Figure 2E from left to right correspond to IMR90 cells treated with combinations of enalapril, a BMP receptor inhibitor (LDN193189, LDN), and BMP4.
