# Supplementary material for: Enalapril mitigates senescence and aging-related phenotypes in human cells and mice via pSmad1/5/9-driven antioxidative genes
Source: eLife. 2025 Aug 28;14:RP104774. doi: 10.7554/eLife.104774 (PMC12393883; doi:10.7554/eLife.104774)
Supplement: Figure 2—figure supplement 1—source data 1. [file elife-104774-fig2-figsupp1-data1.zip › Figure2-figure supplement1-source data1/Figure2-figure supplement1-source data1.pdf]

Figure 2-figure supplement 1, Source Data 1

Figure 2-figure supplement 1A

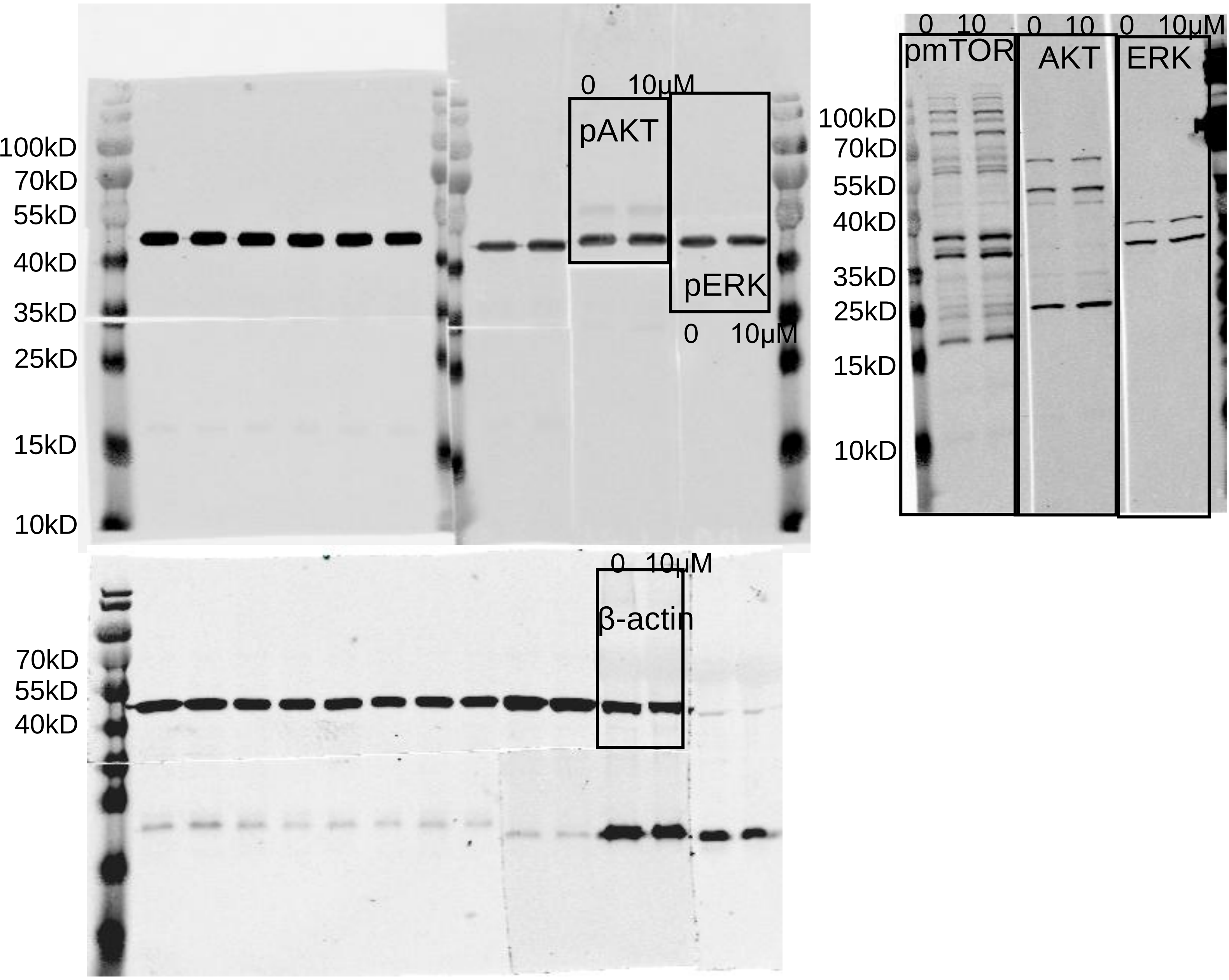

Figure 2-figure supplement 1B

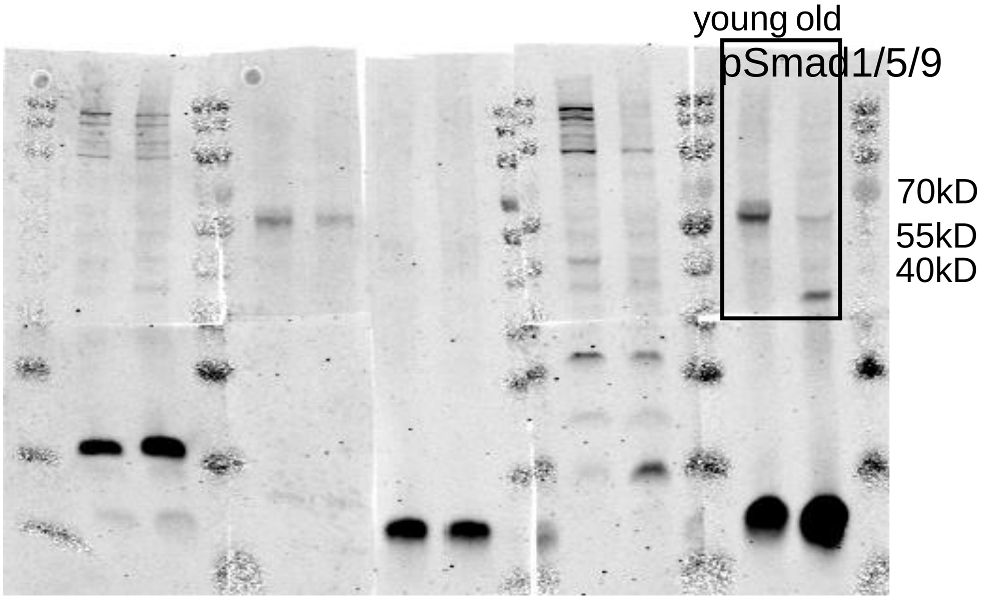

Figure 2-figure supplement 1, Source Data 1

Figure 2-figure supplement 1B

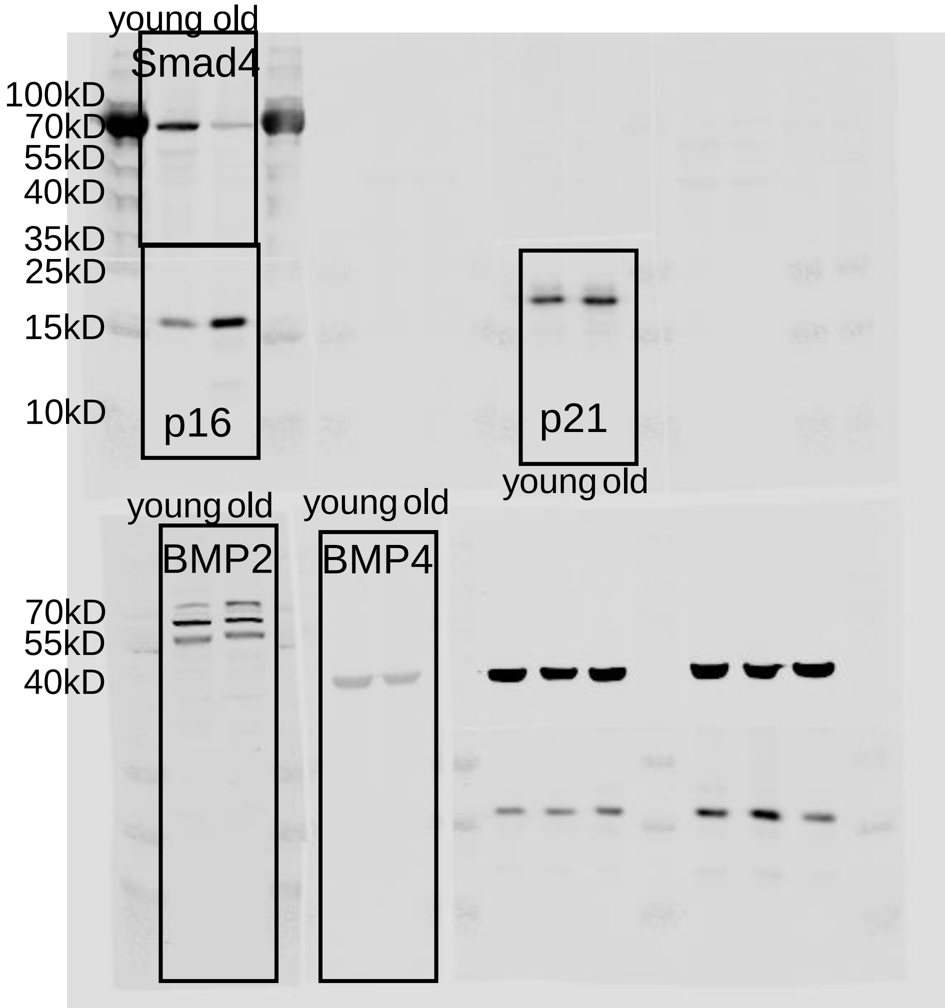

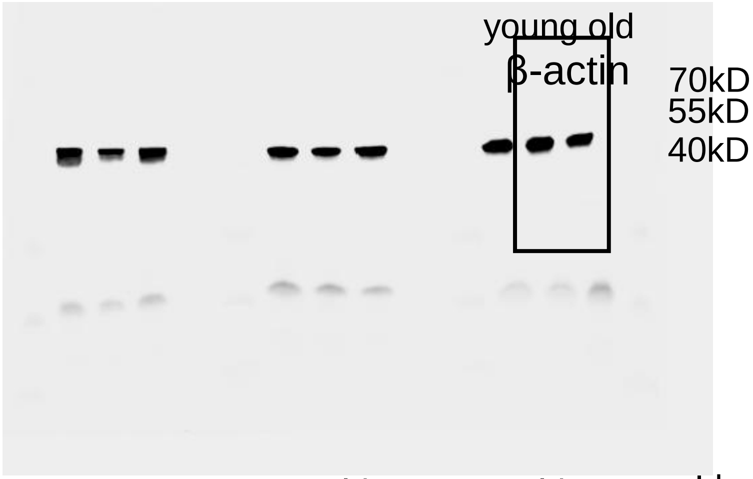

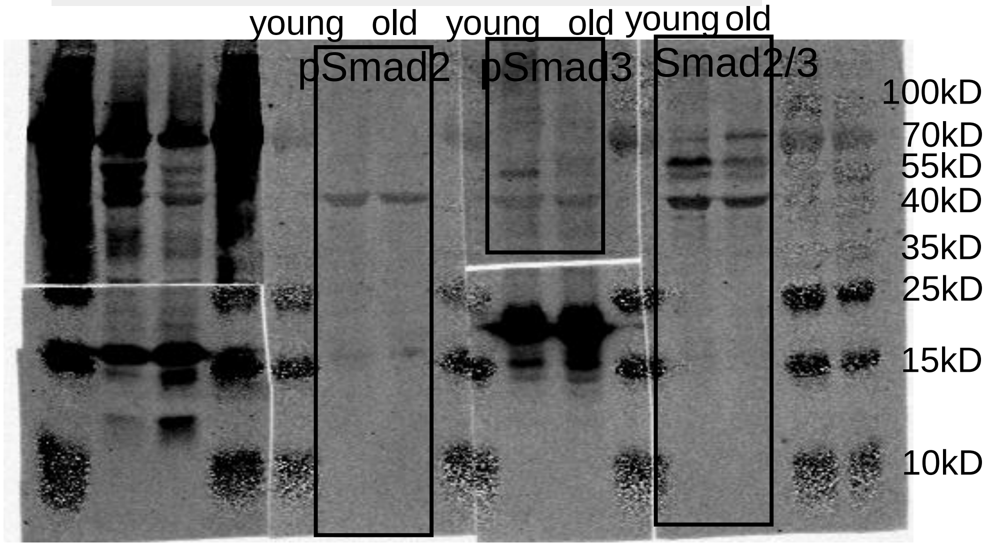

Figure 2-figure supplement 1, Source Data 1. Original membranes corresponding to Figure 2-figure supplement 1A, B. Lanes of Figure 2-figure supplement 1A from left to right correspond to IMR90 cells treated with enalapril at 0 and 10μM, respectively. Lanes of Figure 2-figure supplement 1B from left to right correspond to young and old IMR90 cells, respectively.
