# Supplementary material for: Enalapril mitigates senescence and aging-related phenotypes in human cells and mice via pSmad1/5/9-driven antioxidative genes
Source: eLife. 2025 Aug 28;14:RP104774. doi: 10.7554/eLife.104774 (PMC12393883; doi:10.7554/eLife.104774)
Supplement: Figure 2—figure supplement 3—source data 1. [file elife-104774-fig2-figsupp3-data1.zip › Figure2-figure supplement3-source data1/Figure2-figure supplement3-source data1.pdf]

Figure 2-figure supplement 3, Source Data 1

Figure 2-figure supplement 3A

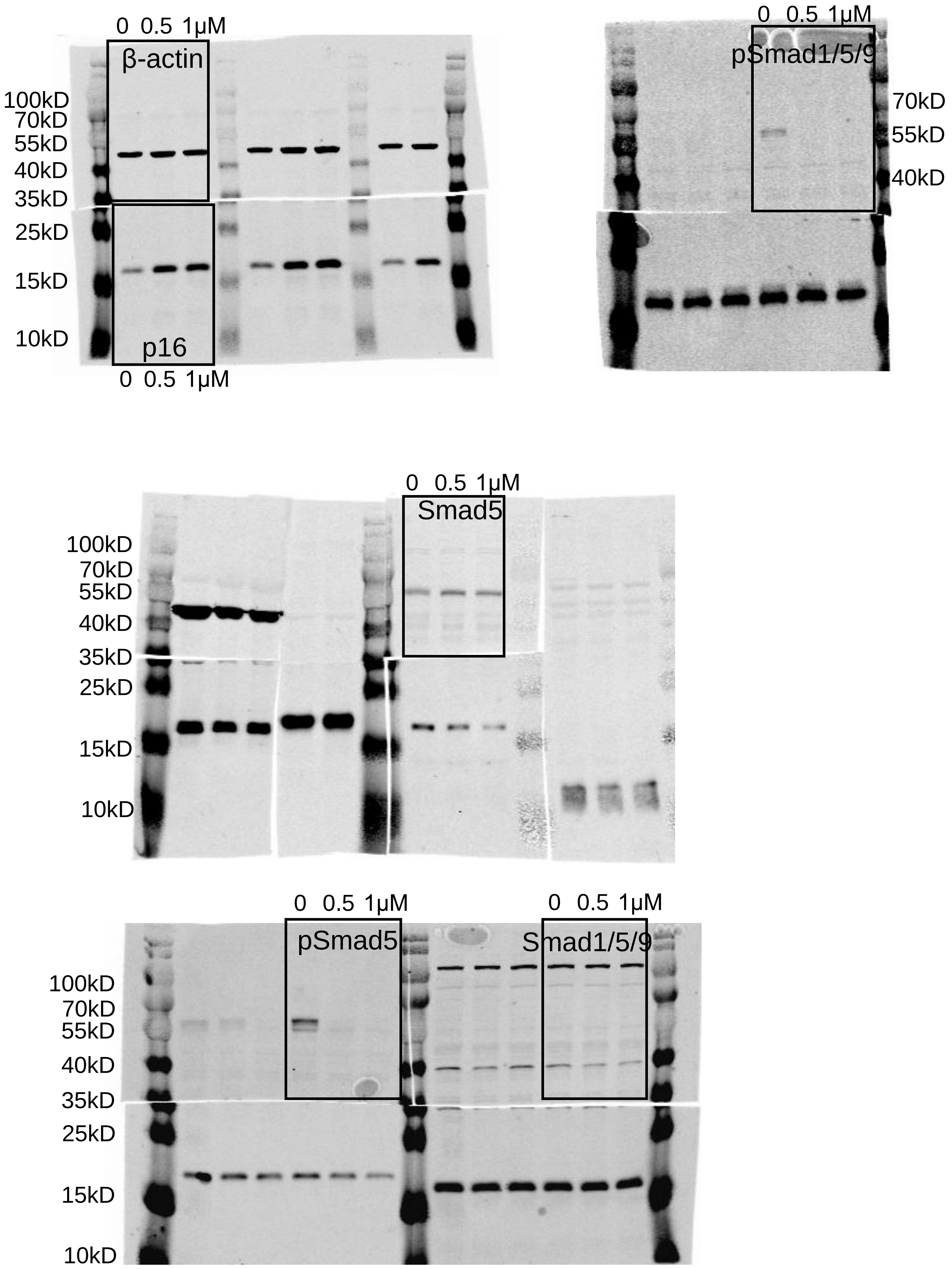

Figure 2-figure supplement 3, Source Data 1

Figure 2-figure supplement 3D

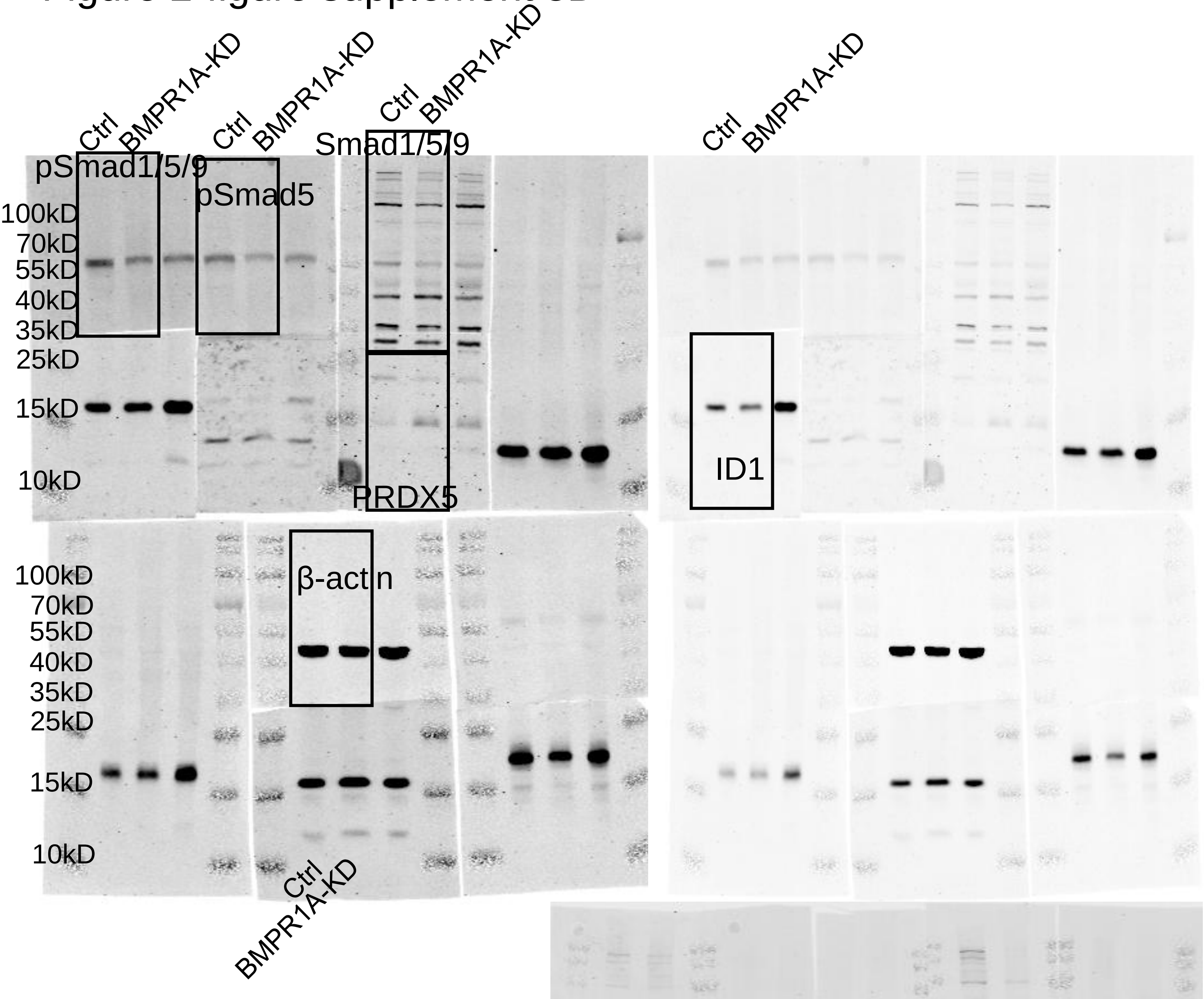

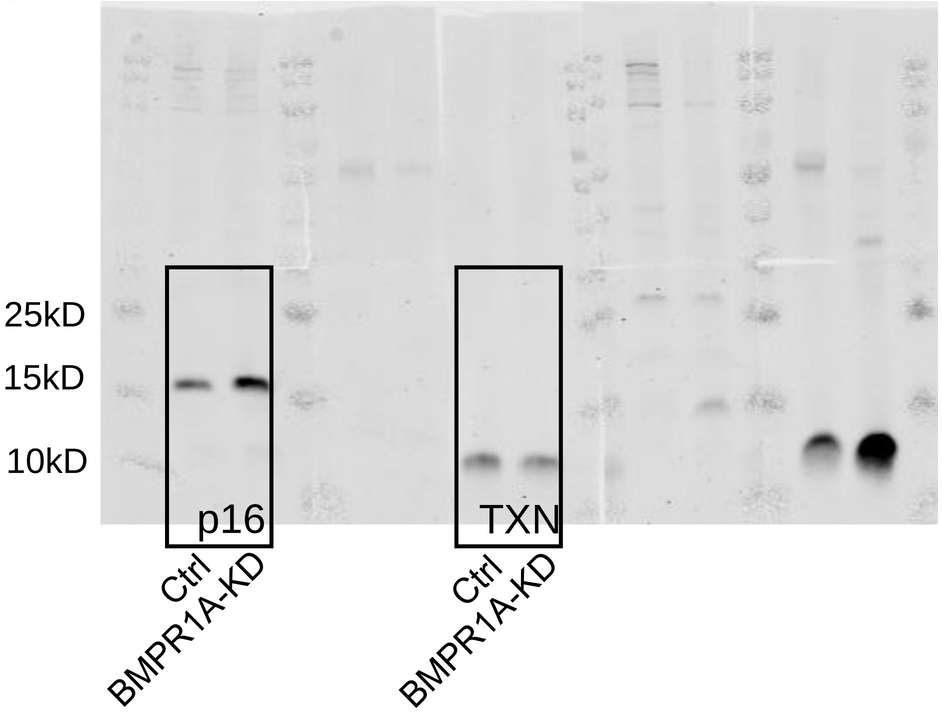

Figure 2-figure supplement 3, Source Data 1. Original membranes corresponding to Figure 2-figure supplement 3A, D. Lanes of Figure 2-figure supplement 3A from left to right correspond to IMR90 cells treated with LDN193189 at 0, 0.5 and 1μM, respectively. Lanes of Figure 2-figure supplement 3D from left to right correspond to Ctrl and BMPR1A knockdown IMR90 cells, respectively.
