# Supplementary material for: Enalapril mitigates senescence and aging-related phenotypes in human cells and mice via pSmad1/5/9-driven antioxidative genes
Source: eLife. 2025 Aug 28;14:RP104774. doi: 10.7554/eLife.104774 (PMC12393883; doi:10.7554/eLife.104774)
Supplement: Figure 3—source data 1. [file elife-104774-fig3-data1.zip › Figure3-source data1/Figure3-source data1.pdf]

Figure 3, Source Data 1

Figure 3C

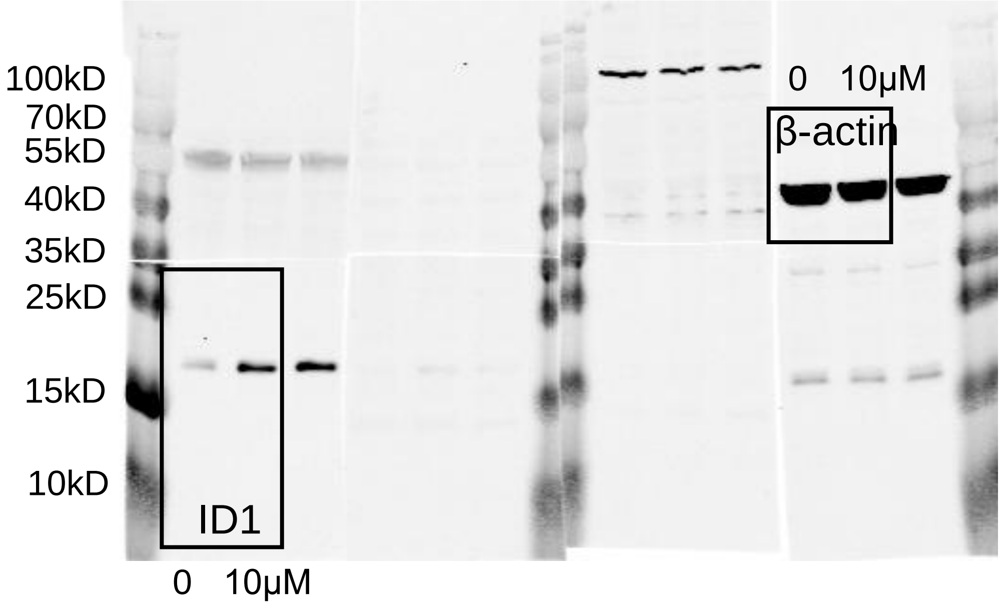

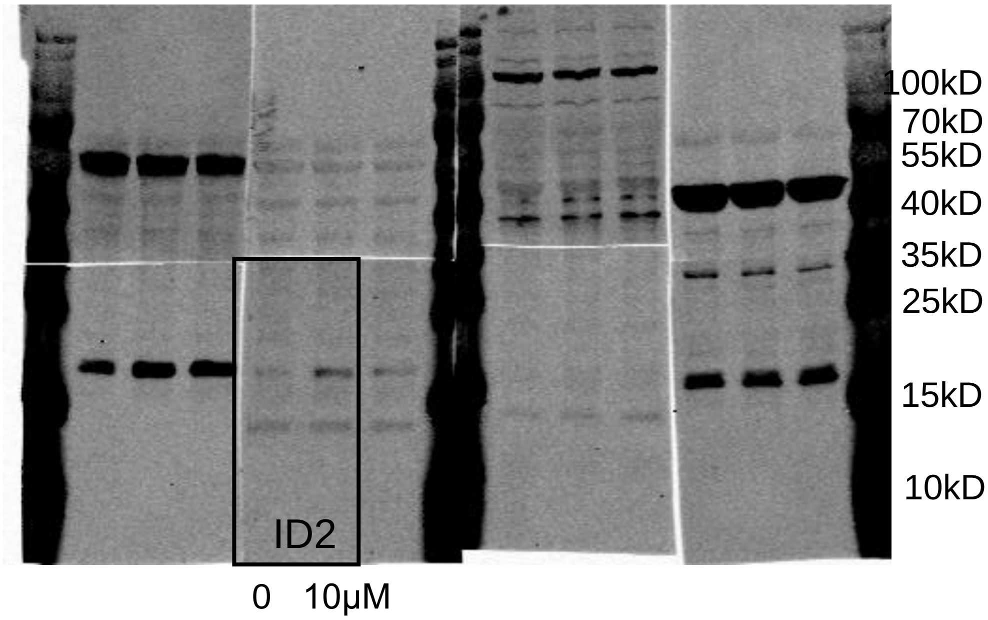

Figure 3, Source Data 1

Figure 3E

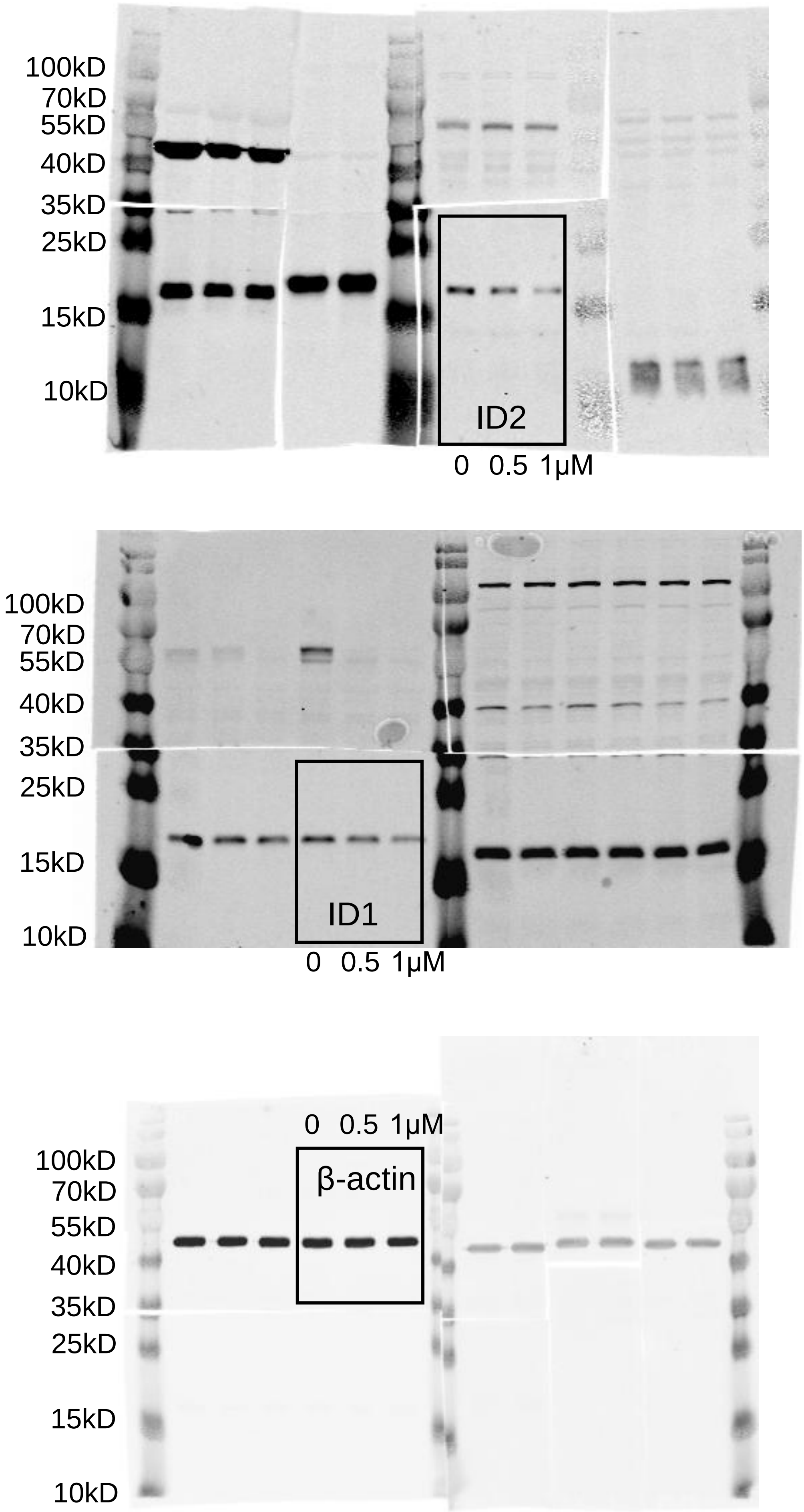

Figure 3, Source Data 1

Figure 3F

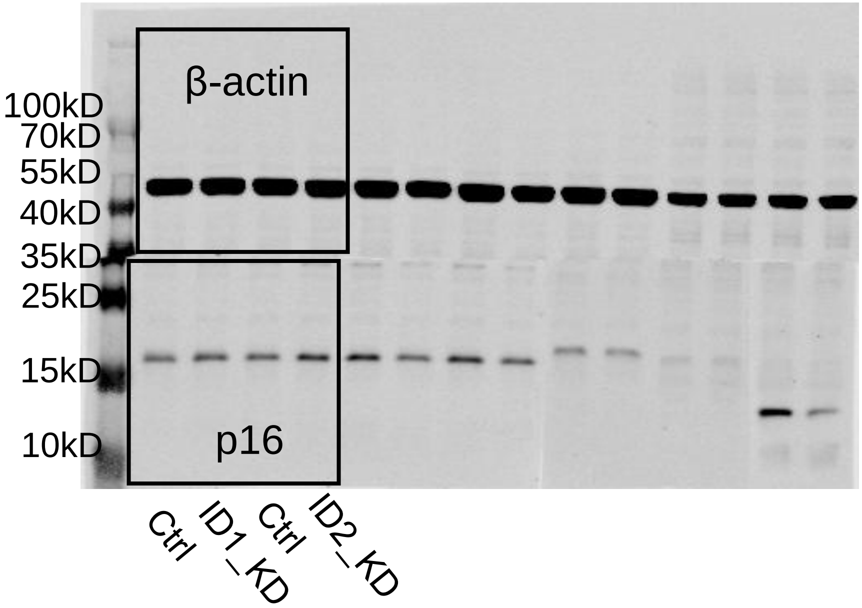

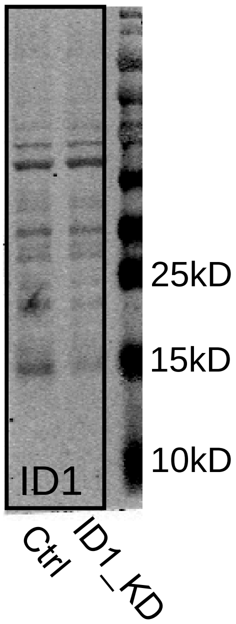

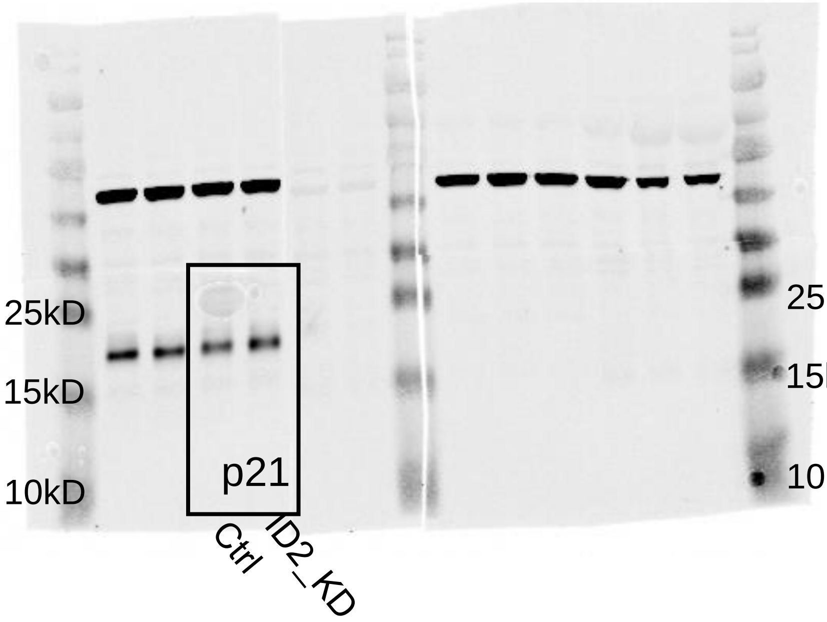

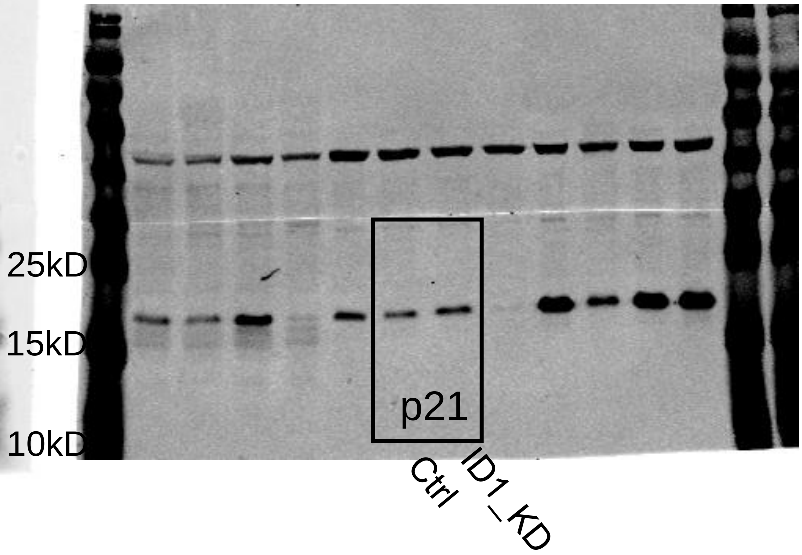

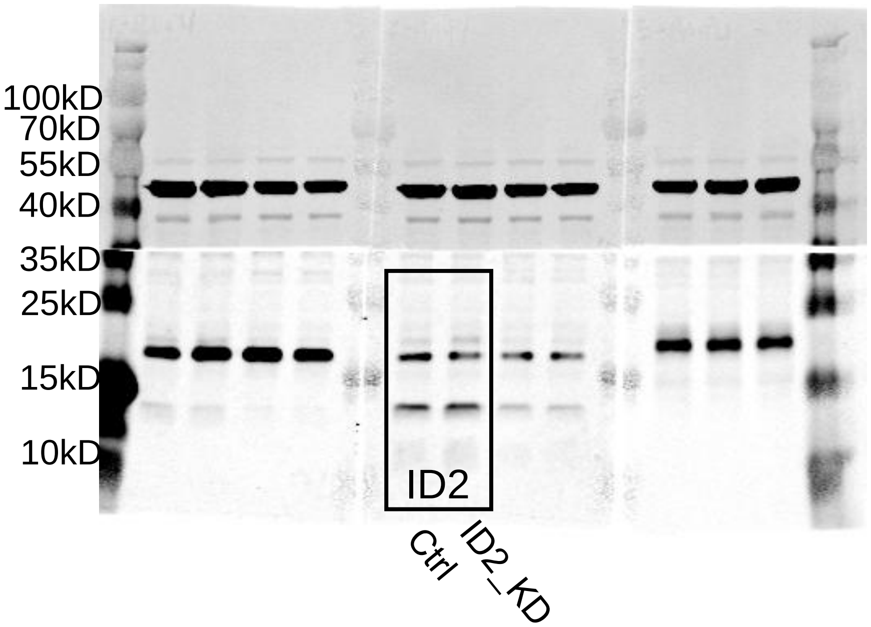

Figure 3, Source Data 1

Figure 3I

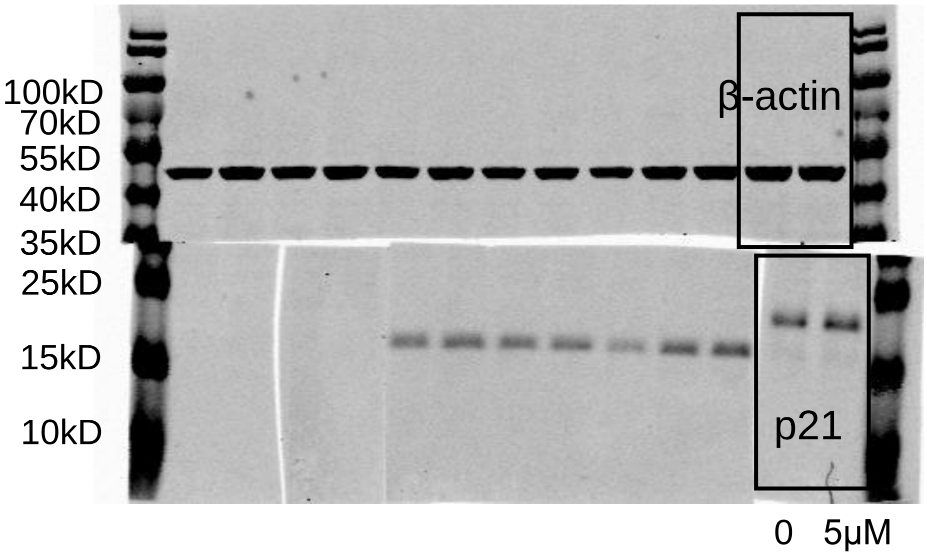

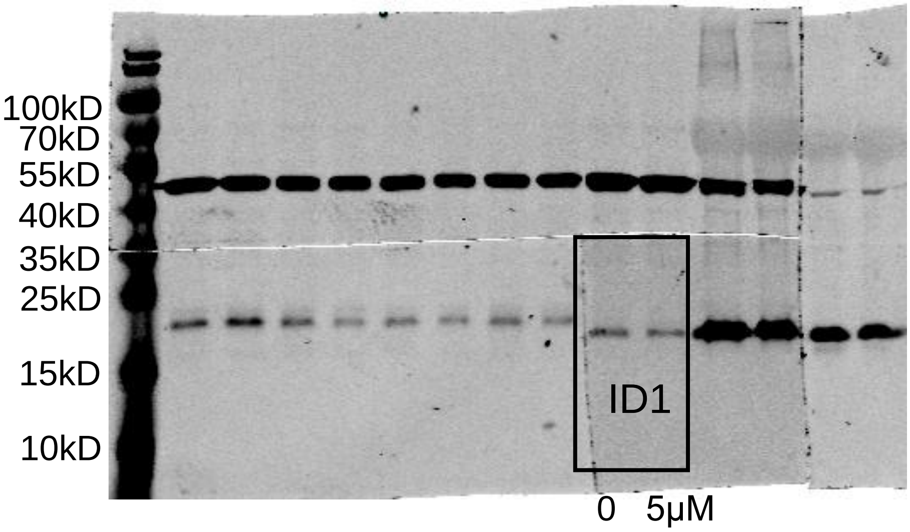

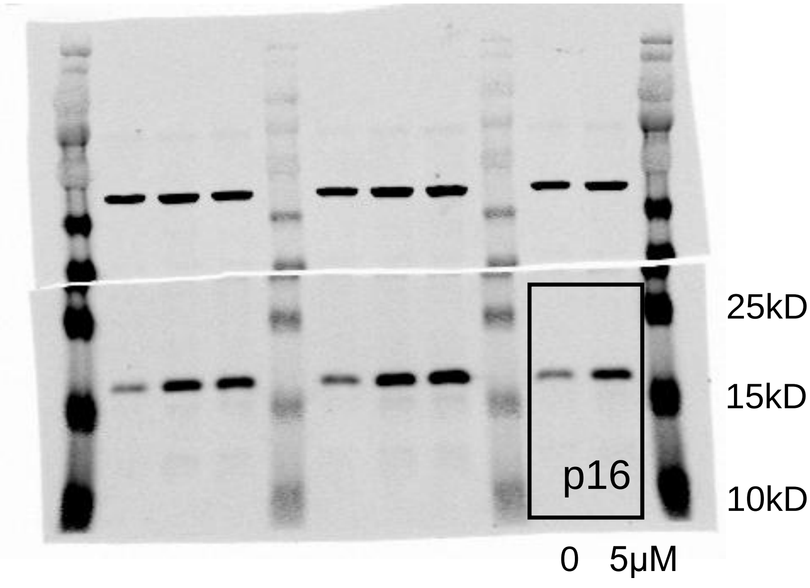

Figure 3, Source Data 1

Figure 3J

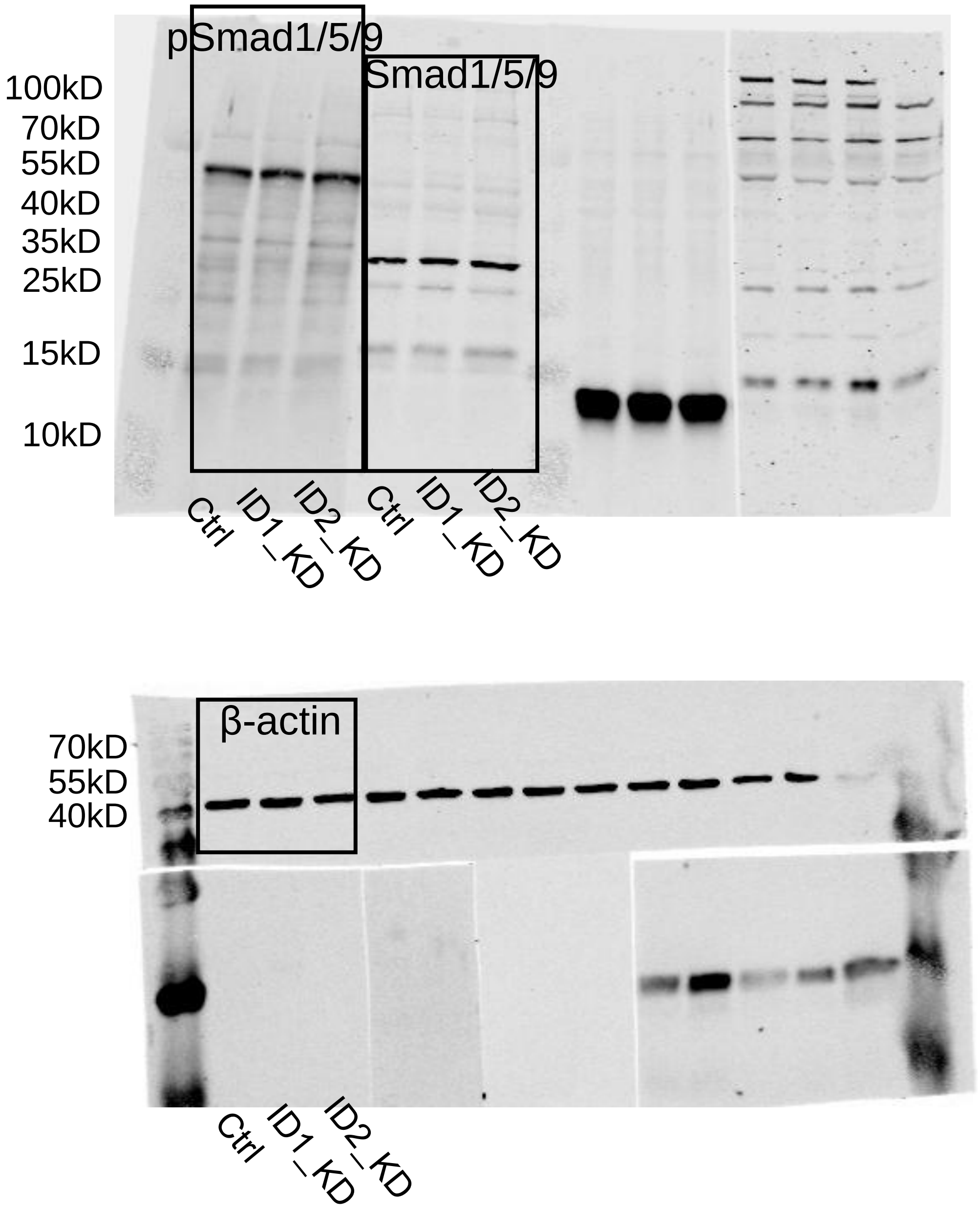

Figure 3, Source Data 1. Original membranes corresponding to Figure 3C, E, F, I and J. Lanes of Figure 3C from left to right correspond to IMR90 cells treated with enalapril at 0 and 10μM, respectively. Lanes of Figure 3E from left to right correspond to IMR90 cells treated with LDN193189 at 0, 0.5 and 1μM, respectively. Lanes of Figure 3F from left to right correspond to Ctrl and ID knockdown IMR90 cells, respectively. Lanes of Figure 3I from left to right correspond to IMR90 cells treated with ID1 inhibitor at 0 and 5μM, respectively. Lanes of Figure 3J from left to right correspond to Ctrl, ID1 knockdown and ID2 knockdown IMR90 cells, respectively.
