# Supplementary material for: Enalapril mitigates senescence and aging-related phenotypes in human cells and mice via pSmad1/5/9-driven antioxidative genes
Source: eLife. 2025 Aug 28;14:RP104774. doi: 10.7554/eLife.104774 (PMC12393883; doi:10.7554/eLife.104774)
Supplement: Figure 4—source data 1. [file elife-104774-fig4-data1.zip › Figure4-source data1/Figure4-source data1.pdf]

Figure 4, Source Data 1

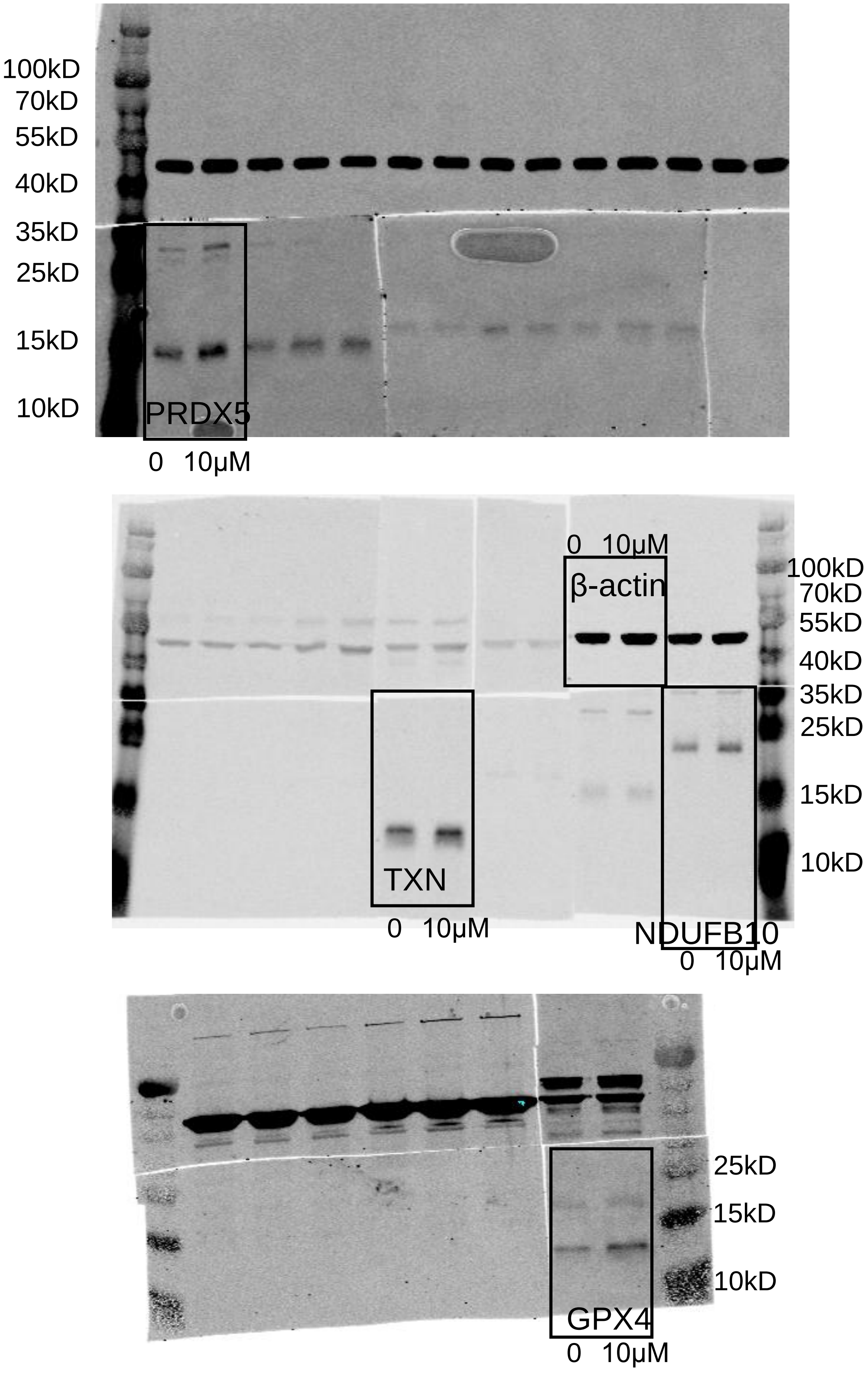

Figure 4, Source Data 1. Original membranes corresponding to Figure 4D. Lanes from left to right correspond to IMR90 cells treated with enalapril at 0 and 10μM, respectively.
