# Supplementary material for: Enalapril mitigates senescence and aging-related phenotypes in human cells and mice via pSmad1/5/9-driven antioxidative genes
Source: eLife. 2025 Aug 28;14:RP104774. doi: 10.7554/eLife.104774 (PMC12393883; doi:10.7554/eLife.104774)
Supplement: Supplementary file 1. [file elife-104774-supp1.docx]

**Supplementary File 1** List of primers used in RT-qPCR

| **Name** | **Primer sequence** |
| --- | --- |
| IL1β | For: ATGATGGCTTATTACAGTGGCAA  Rev: GTCGGAGATTCGTAGCTGGA |
| IL6 | For: ACTCACCTCTTCAGAACGAATTG  Rev: CCATCTTTGGAAGGTTCAGGTTG |
| CXCL10 | For: GTGGCATTCAAGGAGTACCTC  Rev: TGATGGCCTTCGATTCTGGATT |
| CXCL16 | For: CCCGCCATCGGTTCAGTTC  Rev: CCCCGAGTAAGCATGTCCAC |
| CCL2 | For: AAGACCATTGTGGCCAAGGA  Rev: TTCGGAGTTTGGGTTTGCT |
| MMP2 | For: CCCACTGCGGTTTTCTCGAAT  Rev: CAAAGGGGTATCCATCGCCAT |
| BMPR1A | For: CTTTACCACTGAAGAAGCCAGCT  Rev: AGAGCTGAGTCCAGGAACCTGT |
| ID1 | For: CTGCTCTACGACATGAACGG  Rev: GAAGGTCCCTGATGTAGTCGAT |
| ID2 | For: AGTCCCGTGAGGTCCGTTAG  Rev: AGTCGTTCATGTTGTATAGCAGG |
| ID3 | For: GAGAGGCACTCAGCTTAGCC  Rev: TCCTTTTGTCGTTGGAGATGAC |
| ACTB | For: CACCCCGTGCTGCTGAC  Rev: CCAGAGGCGTACAGGGATAG |
| TXN | For: GTGAAGCAGATCGAGAGCAAG  Rev: CGTGGCTGAGAAGTCAACTACTA |
| GPX4 | For: GAGGCAAGACCGAAGTAAACTAC  Rev: CCGAACTGGTTACACGGGAA |
| PRDX5 | For: CTTCACCCCTGGATGTTCCAA  Rev: AGGCATCATTAACACTCAGACAG |
