# Supplementary material for: Enalapril mitigates senescence and aging-related phenotypes in human cells and mice via pSmad1/5/9-driven antioxidative genes
Source: eLife. 2025 Aug 28;14:RP104774. doi: 10.7554/eLife.104774 (PMC12393883; doi:10.7554/eLife.104774)
Supplement: Supplementary file 2. [file elife-104774-supp2.docx]

**Supplementary File 2** List of primers used in pSmad1/5/9 ChIP-qPCR

| **Name** | **Primer sequence** | **Notes** |
| --- | --- | --- |
| HPRT1 | For: TGTTTGGGCTATTTACTAGTTG  Rev: ATAAAATGACTTAAGCCCAGAG | Negative control  ([Morikawa et al., 2011](#_ENREF_2); [Sullivan & Santos, 2020](#_ENREF_3)) |
| HBB | For: GGGCTGAGGGTTTGAAGTCC  Rev: CATGGTGTCTGTTTGAGGTTGC | Negative control  ([Morikawa et al., 2011](#_ENREF_2)) |
| ID1 | For: AGTCCGTCCGGGTTTTATG  Rev: TGTGTCAGCGTCTGAACCAG | Positive control  ([Morikawa et al., 2011](#_ENREF_2); [Sullivan & Santos, 2020](#_ENREF_3)) |
| ID2 | For: ACTCTATTTACCACCCCAGC  Rev: AGCTTCCCTTCGTCCCCAT |  |
| TXN | For: CAGGGCTGGATTCCTCGAAA  Rev: CAAGGACGTACACACCGAGA |  |
| PRDX5 | For: GTATGGGACTAGCTGGCGTG  Rev: TCACTGTACCGTCTTGCTGC |  |
| GPX4 | For: GAAGCAGAGACGGGAGGTTC  Rev: CTTGTGTCTAGGAGGCCGTG |  |
| SOD3 | For: TCTGAGGGGTTAGTGGGGAG  Rev: CCCCTTTCTCGTCACTCCAG |  |

**References**

Morikawa, M., Koinuma, D., Tsutsumi, S., Vasilaki, E., Kanki, Y., Heldin, C. H., . . . Miyazono, K. (2011). ChIP-seq reveals cell type-specific binding patterns of BMP-specific Smads and a novel binding motif. *Nucleic Acids Res, 39*(20), 8712-8727. doi:10.1093/nar/gkr572

Sullivan, A. E., & Santos, S. D. M. (2020). An Optimized Protocol for ChIP-Seq from Human Embryonic Stem Cell Cultures. *STAR Protoc, 1*(2), 100062. doi:10.1016/j.xpro.2020.100062
