# Supplementary material for: Enalapril mitigates senescence and aging-related phenotypes in human cells and mice via pSmad1/5/9-driven antioxidative genes
Source: eLife. 2025 Aug 28;14:RP104774. doi: 10.7554/eLife.104774 (PMC12393883; doi:10.7554/eLife.104774)
Supplement: Supplementary file 3. [file elife-104774-supp3.docx]

**Supplementary File 3** List of shRNA target sequence used in knock down experiment

| **Name** | **Target Sequence** |
| --- | --- |
| shID1 #1 | CCGGCCTACTAGTCACCAGAGACTTCTCG  AGAAGTCTCTGGTGACTAGTAGGTTTTT |
| shID1 #2 | CCGGACTCGGAATCCGAAGTTGGAACTC  GAGTTCCAACTTCGGATTCCGAGTTTTTT |
| shID2 #1 | CCGGCCCTTCTGAGTTAATGTCAAACTCG  AGTTTGACATTAACTCAGAAGGGTTTTT |
| shID2 #2 | CCGGCCCACTATTGTCAGCCTGCATCTCG  AGATGCAGGCTGACAATAGTGGGTTTTT |
| shBMPR1A | CCGGCGCCAATCTCATACAAGCCATCTC  GAGATGGCTTGTATGAGATTGGCGTTTTT |
