# Supplementary material for: Enalapril mitigates senescence and aging-related phenotypes in human cells and mice via pSmad1/5/9-driven antioxidative genes
Source: eLife. 2025 Aug 28;14:RP104774. doi: 10.7554/eLife.104774 (PMC12393883; doi:10.7554/eLife.104774)
Supplement: Supplementary file 4. [file elife-104774-supp4.docx]

**Supplementary File 4** List of antibodies

| **Name** | **Source** | **Number** |
| --- | --- | --- |
| Mouse monoclonal anti-β-actin | Santa Cruz Biotechnology | Cat#sc-47778 |
| Rabbit monoclonal anti-CDKN2A/p16INK4a | Abcam | Cat#ab108349 |
| Rabbit monoclonal anti-p21 | Abcam | Cat#ab109199 |
| Rabbit monoclonal anti-Phospho-SMAD1 (Ser463/465)/ SMAD5 (Ser463/465)/ SMAD9 (Ser465/467) | Cell Signaling Technology | Cat#13820S |
| Rabbit monoclonal anti-SMAD1 + SMAD5 + SMAD9 (phosphor S463 + S465 + S467) | Abcam | Cat#ab92698 |
| Rabbit monoclonal anti-Phospho-SMAD5 (Ser463/465) | Huabio | Cat#ET1605-5 |
| Rabbit polyclonal anti-SMAD1/5/9 | Immunoway | Cat#YT4325 |
| Rabbit monoclonal anti-SMAD5 | Huabio | Cat#ET1606-26 |
| Rabbit monoclonal anti-SMAD4 | Abcam | Cat#ab40759 |
| Rabbit polyclonal anti-SMAD2 (Ser465/467) | Cell Signaling Technology | Cat#3101S |
| Rabbit monoclonal anti-SMAD3 (Ser423/425) | Cell Signaling Technology | Cat#9520S |
| Rabbit polyclonal anti-SMAD2/3 | Cell Signaling Technology | Cat#3102S |
| Rabbit polyclonal anti-BMP2 | Huabio | Cat#ER80602 |
| Mouse monoclonal anti-BMP4 | Huabio | Cat#EM1706-23 |
| Rabbit polyclonal anti-ID1 | Immunoway | Cat#YN0093 |
| Rabbit polyclonal anti-ID2 | Immunoway | Cat#YN2457 |
| Rabbit polyclonal anti-PRDX5 | ABclonal | Cat#A1269 |
| Rabbit monoclonal anti-TXN | ABclonal | Cat#A4024 |
| Rabbit monoclonal anti-TXN2 | ABclonal | Cat#A4424 |
| Rabbit polyclonal anti-NDUFB10 | ABclonal | Cat#A9382 |
| Rabbit monoclonal anti-GPX4 | Abmart | Cat#T56959 |
| IRDy 800CW Goat anti-Mouse IgG Secondary Antibody | LI-COR | Cat#926-32210 |
| IRDy 800CW Goat anti-Rabbit IgG Secondary Antibody | LI-COR | Cat#926-32211 |
